# Influence of curing pressure and surface treatment on mechanical properties of hybrid fiber metal laminates

**DOI:** 10.1038/s41598-026-61551-1

**Published:** 2026-07-17

**Authors:** M. Megahed, A. M. Alsaeedy, A. E. Alshorbagy, M. Atta

**Affiliations:** Department of Mechanical Design and Production Engineering, Faculty of Engineering, Zagazig University, P.O. Box 44519, Zagazig, Egypt

**Keywords:** Fiber metal laminate, Nanoparticles, Hybrid composites, Al-thickness, Pressure, Sheet treatment, Failure analysis, Engineering, Materials science, Nanoscience and technology

## Abstract

This study investigates the effect of key manufacturing parameters and graphene nanoparticle additions on the tensile behavior of fiber metal laminates (FMLs) using a Taguchi-based experimental design. Several manufacturing parameters were considered: laminate configuration (glass fiber and hybrid glass-carbon fiber reinforcement), aluminum surface treatment (chemical treatment and laser surface texturing with scanning spacings of 1 mm and 2 mm), aluminum thickness (0.5, 0.7, and 1.0 mm), graphene nanoparticle content (0, 0.1, and 0.25 wt%), and curing pressure (2, 5, and 7 bar). Eighteen FMLs specimens were fabricated according to the Taguchi orthogonal array and tested under tensile loading. Ultimate tensile strength ($\:{\upsigma\:}$_ult_), tensile modulus (*E*), toughness modulus (U_T_), and failure strain ($\:\epsilon\:$_f_) were evaluated as performance responses. The findings show laminate configuration significantly affects $\:{\upsigma\:}$_ult_, U_T_, and $\:\epsilon\:$_f_, with the all-glass fiber configuration exhibiting superior performance responses. Graphene content and curing pressure had a minimal effect on tensile properties. The optimal parameter combination for $\:{\upsigma\:}$_ult_ and U_T_ involved a glass fiber laminate configuration with chemically treated aluminum, an aluminum thickness of 0.5 mm, 0% graphene, and a curing pressure of 2 bar. Optimal parameters for *E* include a laser 1 mm scanning texture, glass fiber laminate configuration, aluminum thickness of 0.5 mm, 0% graphene nanoparticles, and a curing pressure of 5 bar. Additionally, optimal parameters for $\:\epsilon\:$_f_ are glass fiber configuration, chemical surface treatment, aluminum thickness of 1 mm, 0% graphene, and curing pressure of 2 bar. Validation tests indicated the model’s predictions were accurate, with prediction errors under 5%, highlighting its statistical reliability.

## Introduction

Fiber Metal Laminates consist of alternating layers of metal sheets and glass reinforcement/epoxy composites. They are considered super hybrid composites that are cured under specific pressure and temperature conditions^1^. When compared to monolithic metal sheets, combination of distinctive properties of composites and metals offer a greater variety of options for their applications in the automotive industries and aerospace^2^. Fiber metal laminates have several advantages such as low density, high specific strength, excellent damage tolerance, fatigue resistance and impact resistance^3^. At present, aluminum alloy has become the primary metal base in fiber metal laminates (FMLs), and two generations of typical fiber reinforced aluminum alloy laminates, aramid fibers reinforced aluminum alloy laminates (ARALL) and glass fiber reinforced aluminum alloy laminates (GLARE) have been developed successfully^4^. The influence of the initial condition of the metal substrate has been found to be significant in the adhesion of the fiber/epoxy prepreg composite to the metal substrate after cure. The initial condition of the metal substrate has been found to be controlled by the surface treatment process. Thus, the surface treatment of aluminum alloys has received considerable research attention^5^.

A crucial factor that influences the characteristics of FMLs is the interfacial adhesion between the composite material and the metal layers. In fact, the interface of FMLs is a complicated discontinuous system composed of metals, matrixes and fibers. The strength, fatigue behavior and other properties of the FMLs are strongly related to characteristics of the interface^6^. In general, these methods can be classified as mechanical, chemical, electrochemical, coupling agents, etc. Surface treatment methods used prior to lamination can greatly affect the surface morphology, surface energy and wettability of metals and thus the mechanical properties of FMLs^7^. In fact, surface treatment methods can improve the mechanical properties of adhesive bonding through different mechanisms. The high-roughened surface of metals, caused by mechanical methods, facilitates enhanced bonding performance through the increase in surface area for bonding^6^.

Liu et al.^8^ classified surface treatment methods into three main groups: mechanical treatments (sandblasting, mechanical abrasion), chemical treatments (oxidation, etching), and physical treatments (laser forming, plasma treatment). Research has shown that multi-step surface treatments that combine mechanical and chemical methods achieve superior bonding strength compared to single-step treatments. Belkondi et al.^9^ investigated the effect of different treatments on interfacial adhesion strength and failure modes of carbon fiber reinforced aluminum laminates (CARALL). This comprehensive study evaluated mechanical wear, nitric acid etching, P2 etching, sulfuric acid oxidation (SAA), and electric discharge machining, EDM formation. The results showed that SAA treatment increased the T-peel strength by 320.5% and the shear strength by 342.1% compared to the untreated samples. Furthermore, Caggiano et al.^10^ revealed that chemical treatments such as electrochemical oxidation create nanoporous structures that enhance the mechanical interlocking between the aluminum and composite layers. The study indicated that oxidation at a temperature of 40 degrees Celsius produces homogeneous oxide layers resembling a honeycomb with pore diameters ranging from 100 to 200 nanometers, resulting in a 317.2% improvement in apparent shear strength. Moreover, Alshamma and Kadhim^11^ improved the delamination resistance in GLARE using various surface preparation techniques, including chemical etching and laser treatments. Their work showed that surface roughness through chemical etching significantly improves interfacial adhesion by increasing the surface area and providing mechanical anchoring sites for the polymer matrix. Besides, recent advances in laser surface treatment have been promising. Liu et al.^12^ reported that laser irradiation is a viable surface preparation method, as it increases mechanical interlocking while ultimately improving adhesion strength at the interfaces. Balkundhi et al.^9^ analyzed traditional and non-traditional methods of surface treatment and found that laser treatments were one of the more appealing and successful methods to enhance peel strength properties at FMLs interfaces; electro-etching improved the outcomes even further, particularly when shear strength properties were analyzed. Caggiano et al.^10^ considered several deployment methods as a part of the study, including polishing, laser engraving, sandblasting, and chemical etching, and found that laser engraving increased surface roughness while providing better wettability; care should be taken with their parameters, however, to avoid the introduction of excessive concentrations of stress.

Additionally, the improved mechanical and physical characteristics of nanocomposites over their micro composites counterparts have attracted a lot of interest lately. Because of their comparatively huge surface area per unit volume and nanoscale size, nanofillers exhibit important features. Enhancing the mechanical characteristics of FMLs involves adding nano reinforcing to the polymeric matrix^13–15^. The incorporation of nano-graphite particles into FMLs matrix structures has emerged as an effective method to enhance mechanical properties by improving interfacial bonding and strengthening the matrix^16–19^. Numerous investigations have shown that substantial improvements in the mechanical performance of polymer composites can be achieved with very little graphene nanoplatelets (GNP). GNP can substantially improve stress transfer between the matrix and reinforcement at low weight fractions because of their very high aspect ratio, huge specific surface area, and superior intrinsic mechanical characteristics^20,21^.

Turaka and Bandaru^22^ studied the mechanical performance of glass fiber/polymer composites by adding multi-walled carbon nanotubes (MWCNT) and graphene nanoparticles and their hybrid effect at different weight percentages from 0.1 to 0.3%. The results showed a substantial improvement with the 0.2% hybrid combination of MWCNT and GNP which showed 80% and 74% in the compressive strength and compressive modulus, respectively. More than 0.2% of fillers, the properties started to degrade due to the agglomeration of MWCNT and GNP. Moreover, Turaka et al.^23^ reported glass fibre/epoxy composite blended with MWCNT/graphene by 0.2 wt% has shown higher fatigue life by 56% and higher tensile strength by 36%. In addition, by incorporating only 0.3 wt% GNPs into the neat epoxy system, the average tensile strength, tensile modulus, and tensile strain of the composites were significantly enhanced by 71.62%, 16.30%, and 47.45% compared to neat epoxy^24^. Furthermore, an enhancement of the 8.96% in tensil strength and 31.18% in flexurl strength were observed with coating of 0.2 wt% MWCNT and GNP added to hybrid Kevlar/Carbon/epoxy composites^25^. The influence of incorporation of graphene nanoplatelets on quasi-static behavior of composite and fiber metal laminate panels was investigated^26^. The results showed that adding 0.2 wt% GNP improved the strength and fracture toughness of specimens by delaying the failure modes. Generally, researchers have found that GNP loading range from 0.1 to 0.3 weight%, significantly improve strength, stiffness, and interlaminar characteristics. However, nanoparticle agglomeration frequently occurs when the GNP content is increased over the ideal threshold.

Likewise, the arrangement and type of reinforcing fibers in fiber metal laminates have a large impact on their mechanical properties. Recently, it has been shown through testing that adding additional fiber types can positively provide the laminate better performing characteristics. The hybrid configuration with glass and carbon fibers has been getting significant interest^27^. Zhang et al.^28^ studied the mechanical stacking system’s properties of hybrid composites reinforced with hybrid reinforcement and showed that a hybrid composite sheet containing 50% carbon is the optimal for tensile stress properties. Furthermore, Vo et al.^29^ studied the effect of hybridization of carbon-glass fiber reinforced aluminum laminates and found that careful placement of carbon fibers in the outer layers and glass fibers in the inner layers, resulted in improved tensile strength and impact resistance over the use of single fibers alone. The study revealed that hybrid configurations could experience as much as 35% greater specific strength than monolithic glass fiber reinforced aluminum laminates (GLARE). In addition, the thickness of the aluminum layers in FMLs is particularly important for the overall mechanical behavior of the laminate structure as a whole. More studies have recently been done to gain better understanding of the thickness range for various applications of fiber metal laminates. Cortes and Cantwell^30^ demonstrated that thinner aluminum layers (0.2–0.5 mm) had better specific strength properties while thicker layers (1.0–1.5 mm) had better damage tolerance properties. It was determined for aerospace applications, the best thickness to use was between 0.4 and 0.8 mm which uses the most weight efficient thickness while retaining structural integrity. Moreover, Seyed Yaghoubi and Liaw^31^ indicated that impact energy absorption increased, when increasing aluminum thickness from 0.3 mm to 1.2 mm, in the order of 45%. However, the authors indicated that increasing aluminum thickness beyond 1.0 mm, meant additional weight was carried with little performance increase.

Manufacturing pressure is among the most important processing parameters in the fabrication of fiber metal laminates, impacting consolidation quality, interfacial bonding, void formation, and, ultimately, the mechanical properties of the final composite structure. During curing/lamination, sufficient consolidation pressure is necessary to press the layers together and enable the trapped air and/or volatiles to escape, reducing void content^10^. Incorrectly applying pressure during the manufacturing process would result in significantly weaker interface bond strength between aluminum and composite layers^32^. If the autoclave pressure or molding press pressure is inadequate, there will be substantially more porosity throughout the laminate^33^. Ostapiuk et al.^34^ systematically investigated the impacts of varying autoclave pressure (0.1, 0.2, and 0.4 MPa) on the eventual properties of FMLs and showed that, at pressures lower than 0.4 MPa, porosity can develop throughout the volume of the polymer composite. Balkundhi et al.^9^ cured Carbon Fiber Reinforced Aluminum Laminates at 4 bar (0.4 MPa) and demonstrated that adequate pressure forces resin into surface textures to form mechanical interlocks. Müller et al.^35^ manufactured GLARE panels using 6 bar autoclave pressure and noted that reduced-pressure routes caused 30–60% decreases in interlaminar shear strength. However, compression molding processes typically operate at lower pressures than autoclave curing. Furthermore, Chen et al.^33^ confirmed that consolidation pressures around 0.4 MPa are necessary to minimize void content in FMLs. Therefore, the range of pressure in this study is chosen to be from 2 to 7 bar to include pressures below, around, and above the critical 0.4 MPa threshold reported in the literature. Additionally, the influence of curing pressure on compression molding techniques, not autoclave pressure on the interfacial bonding and mechanical performance of fiber metal laminates, has not been comprehensively investigated in literature. Establishing the relationship between consolidation pressure, interfacial adhesion, and resulting mechanical properties remains a significant challenge in the development and optimization of FMLs structures.

Despite the extensive research on fiber metal laminates, limited attention has been given to the impact of curing pressure on their tensile performance, especially when combined with other manufacturing variables. Additionally, there are few studies that compare chemical surface treatment with advanced mechanical surface modification methods like laser texture. Furthermore, the combined effects of surface treatment, laminate arrangement, aluminum thickness, curing pressure, and graphene nanoplatelet reinforcement have not been studied. Moreover, in order to maximize the tensile properties of enhanced fiber metal laminates, the combined evaluation of these parameters utilizing a Taguchi–ANOVA approach is investigated. In order to close this gap, the current study will examine how important manufacturing factors interact with FML tensile performance and determine the best processing conditions for improved mechanical qualities. Additionally, the study establishes relationships between processing parameters, tensile behavior, and fracture causes and offers a comparative evaluation of chemical and laser surface treatments.

## Methodology

### Experimental design

Setting the required number of experiments needs to state the influencing variables, which affected the FMLs properties and their levels. From the previous survey, the variables used in this study could be summarized as structure and manufacturing variables. The structure variables included the type of composite laminates and their arrangement, the thickness of the sheet metal, and the percentage of nano-fillers. However, the manufacturing parameters included the manufacturing pressure and the manner of treating the metal sheets’ contact surface. Both mechanical and chemical treatments were applied to sheet metal. The mechanical treatment involves two-dimensional laser machines (X 1 mm, X 2 mm). These five variables were changed to study their effect on the properties of FMLs as shown in Table 1.

**Table 1:** The factors used and their levels.

Parameters	Unit	Level 1	Level 2	Level 3
Configuration	–	10 g	1c + 8 g + 1c	–
Treatment	–	Chemical	Laser X 1 mm	Laser X 2 mm
Thickness	mm	0.5	0.7	1
Nano percentage	%	0	0.10	0.25
Pressure	bar	2	5	7

g, glass fiber; c, carbon fiber.

This design required 162 full factorial experiments. Taguchi created the orthogonal arrays (OAs) to reduce this number of experiments^29^. The applied OA for three levels and five parameters was L_27_ (3^5^) and was eliminated to 18 specimens, according to two laminates order instead of three. The manufacturing parameters for different specimens were shown in Table 2.

In the present study, five control factors were considered. Four factors, namely surface treatment, aluminum thickness, graphene nanoparticle content, and curing pressure, were investigated at three levels, while laminate configuration was investigated at two levels. The configuration factor was limited to two levels as the objective was to compare the two laminate configurations considered in this work: the all-glass fiber metal laminate and the hybrid glass/carbon fiber metal laminate. Therefore, a third configuration level was not included because it was outside the scope of the experimental design. Based on the mixed-level nature of the selected factors, an appropriate Taguchi orthogonal array was selected to accommodate one two-level factor and four three-level factors while maintaining a reduced and systematic experimental matrix compared with a full factorial design. A full factorial design would require a considerably larger number of experiments, increasing material consumption, fabrication time, and testing effort. Therefore, the selected Taguchi OA was used to efficiently evaluate the main effects of the investigated parameters.

**Table 2:** The manufacturing parameters for different specimens.

Experiment number	Configuration	Treatment	AL–thickness (mm)	Nano percentage (%)	Pressure (bar)
1	10 g	Chemical	0.5	0	2
2	10 g	Chemical	0.7	0.1	5
3	10 g	Chemical	1	0.25	7
4	10 g	Laser X 1 mm	0.5	0	5
5	10 g	Laser X 1 mm	0.7	0.1	7
6	10 g	Laser X 1 mm	1	0.25	2
7	10 g	Laser X2 mm	0.5	0.1	2
8	10 g	Laser X2 mm	0.7	0.25	5
9	10 g	Laser X 2 mm	1	0	7
10	1c+8 g+1c	Chemical	0.5	0.25	7
11	1c+8 g+1c	Chemical	0.7	0	2
12	1c+8 g+1c	Chemical	1	0.1	5
13	1c+8 g+1c	Laser X1 mm	0.5	0.1	7
14	1c+8 g+1c	Laser X1 mm	0.7	0.25	2
15	1c+8 g+1c	Laser X 1 mm	1	0	5
16	1c+8 g+1c	Laser X 2 mm	0.5	0.25	5
17	1c+8 g+1c	Laser X 2 mm	0.7	0	7
18	1c+8 g+1c	Laser X 2 mm	1	0.1	2

### Materials

The fabricated FMLs specimens used through this work consisted of skin material and 10 plies of fiber composite as core laminate. Skin material was made of 1050 Aluminum sheet with three different thickness; 0.5, 0.7 and 1 mm, which was supplied by Metallurgical Industries Company, Egypt. Aluminum 1050 was selected as the metallic layer in the present FMLs system due to its low density, excellent corrosion resistance, high ductility, and good formability^36^. Because of these qualities, it is a good material for lightweight structural applications and a good choice for researching how surface treatment and processing parameters affect interfacial bonding. Furthermore, its high ductility promotes progressive failure and energy absorption, which are desirable features in fiber metal laminates intended for aerospace and transportation applications. In addition, the relatively aluminum surface responds effectively to chemical and laser surface treatments, facilitating the study of adhesion mechanisms between the metal and composite layers.

The employed E-glass was woven fiber which possessed density of 300 g/m^2^ and thickness between 0.22 and 0.28 mm, while the carbon fibers were constructed from woven T300 carbon fiber material with a surface density of 230 g/m^2^ and thickness 0.25 mm. Both the glass and carbon fiber fabrics were plain-woven in architecture, in which the warp and weft yarns were interlaced alternately in an over-under pattern, offering improved dimensional stability and balanced mechanical qualities. Throughout the laminate structure, every layer of fiber fabric was positioned in a 0°/90° orientation. The matrix material was epoxy which comprised of part A, Biresin R CR82 (resin), and part B, CH80-6 (hardener), provided by Sika Industry. For some specimens, nano-fillers were incorporated in epoxy with a weight% of 0.1% and 0.25%. These nano-fillers were graphene nanoplatelets of size 5 μm in width, 6–8 nm in thickness, and 120–150 m^2^/g in surface area, which were supplied by Sigma-Aldrich. Chemicals, which were used in sheet treatment, including sodium hydroxide, acetone and hydrochloric acid were supplied by El Nasr Pharmaceutical Chemicals, Egypt.

### Specimen preparation

#### Surface treatment of aluminum sheets

Bonding between the polymeric composite laminate and metal layers is critical for the overall performance of FMLs so suitable surface treatment of the metal layer is required. The first step to producing different specimens was sheet preparation. There were two methods used for surface treatment of aluminum: the first method was chemical treatment, and the second method is Laser surface machining with 2 different spacings.

##### Chemical treatment

**Fig. 1:**
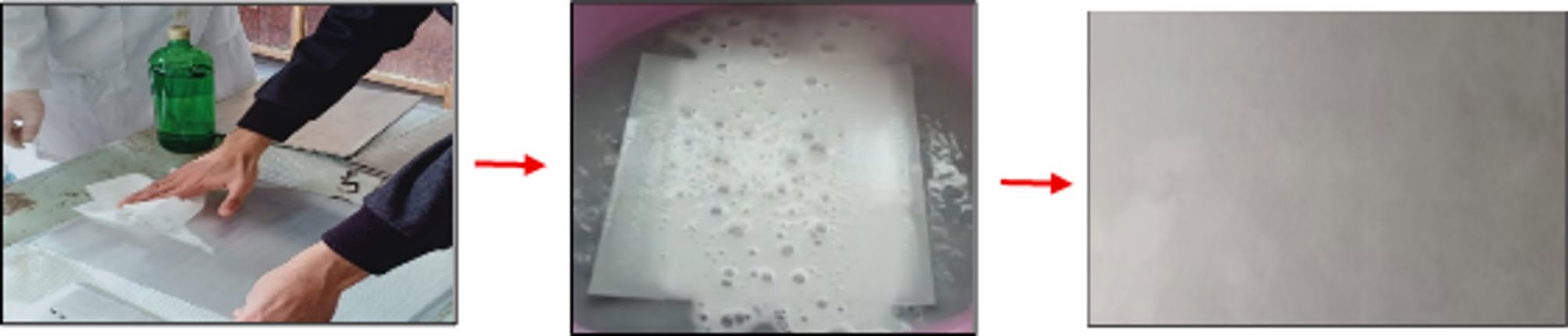
Chemical treatment of aluminum sheets.

Hydrochloric acid (HCl) etching was used to treat the surface, and sodium hydroxide (NaOH) was used for alkaline etching. Acid-alkaline treatment of aluminum sheets results in a porous oxide layer on the surface from the alkaline treatment as well as high roughness from the acid washing. Abrasive sandpapers, number 400, were used to abrade the aluminum sheet’s surfaces, then cleaned with acetone and distilled water. Therefore, to roughen the surfaces, aluminum sheets were submerged in an 11% HCl acid solution for 30 min. Lastly, aluminum sheets were desiccated after being bathed in a 5 wt% NaOH solution at 70 °C for five minutes and cleaned with distilled water. Aluminum sheets were cleaned with acetone and distilled water after 400-grit sanding. After roughening surfaces in an 11 vol% HCl acid solution for 30 min, sheets were bathed in a 5 wt% NaOH solution at 70 °C for five minutes, cleaned with distilled water, and dried. These parameters are chosen from FML surface treatment literature. Park et al.^37^ used HCl-based etching at 10 vol% for 20 min to fabricate GLARE, achieving Ra increases from 0.3 to 1.8 μm. Droździel-Jurkiewicz et al.^38^ found that acid etching at 10–30 min at 5–15 vol% produces micro-scale roughness for mechanical interlocking without excessive material dissolution, following ASTM D2651 guidance. Gonzalez-Canche et al.^39^ assessed NaOH treatments at 10 wt% and 80 °C and found that 5 wt% at 70 °C for 10 min improved surface cleanliness, roughness, and wettability. Caggiano et al.^10^ found that alkaline treatment at 60–80 °C creates nanoporous oxide structures (100–200 nm pore diameter) that serve as mechanical anchoring sites and chemical bonding platforms, with 70 °C optimally balancing oxide formation and surface integrity preservation. This dual acid-alkaline protocol follows Najafi et al.^40^ who used the same sequence to prepare aluminum surfaces in FMLs for marine applications. Figure 1a shows the aluminum surface after treatment as shown in Fig. 1.

##### Laser surface machining of Al sheet

**Fig. 2:**
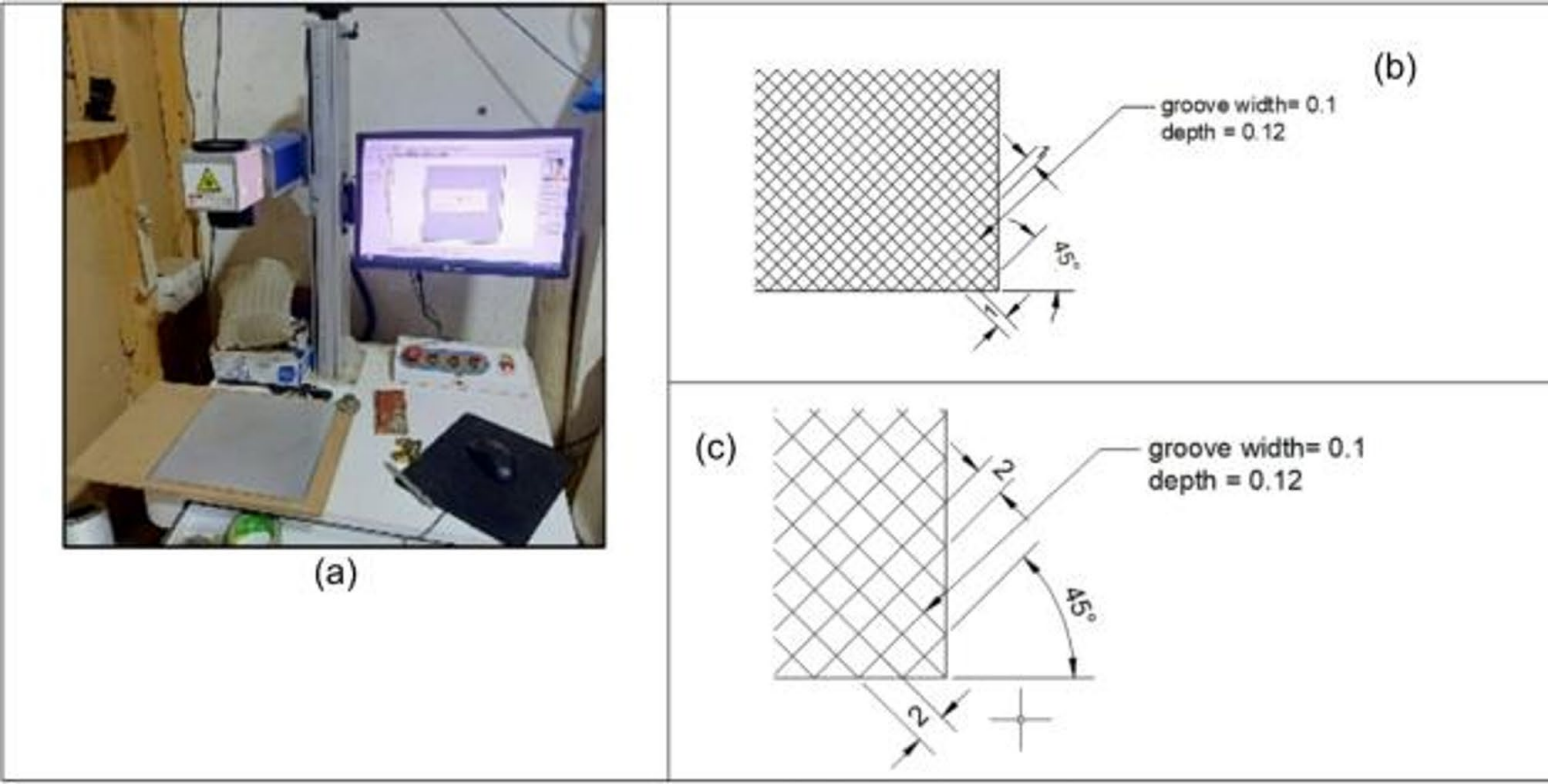
Laser treatment process of AL sheet (**a**) laser beam marking machine, (**b**) laser mark distance of 1 mm pattern and (**c**) laser mark distance of 2 mm pattern.

Aluminum sheets were laser treated with a Wuxi Raycus fiber laser marking machine (RFL-P30 QS, China) with a nominal maximum power of 30 W and a wavelength of 1064 nm was employed (Fig. 2a). All aluminum substrates were cleaned in acetone before laser treatment in order to remove any surface contaminants and greasy residues. The laser interaction zone material is heated above its melting point during nanosecond pulsed laser processing. A portion of the molten aluminum is ejected from the ablation crater, while the rest resolidifies at the groove edges, forming recast layers with melt attachments along the groove walls and rims and scattered micro-pores. Post-processing cleaning included compressed air removal of loose debris and ablation residue. After laser texturing, no aggressive chemical cleaning or oxide removal was done to remove the surface features that provide the mechanical interlocking adhesion mechanism. Subsequently, a cross-hatched X-texture pattern was engraved by two sets of orthogonal parallel grooves at an angle of 45° with inter-groove spacings of 1 mm and 2 mm, respectively, as shown in Figures (2b and 2c). Ten passes were made over each pattern to achieve a rough enough surface topography to encourage strong interfacial adhesion. The groove geometry obtained was characterized by an approximate depth of 0.12 mm, a width of approximately 0.1 mm, and surface coverage ratios of about 55% and 30% for the 1 mm and 2 mm spacing configurations, respectively. The main adhesion mechanism facilitated by this texturing technique is mechanical interlocking. The adhesive or bonding agent penetrates and locks into the grids of the grooves physically formed. Table 3 summarizes the full machining parameters for the laser treatment operation.

**Table 3:** Laser treatment, machining parameters.

Average power (W)	Processing speed (mm/s)	Pulse frequency (kHz)	Pulse duration (ns)	Spot diameter (mm)	Focused spot diameter (mm)	Pulse energy (mJ)
30	250	20	150	6–8	0.1	1.5

#### Fiber metal laminate preparation

**Fig. 3:**
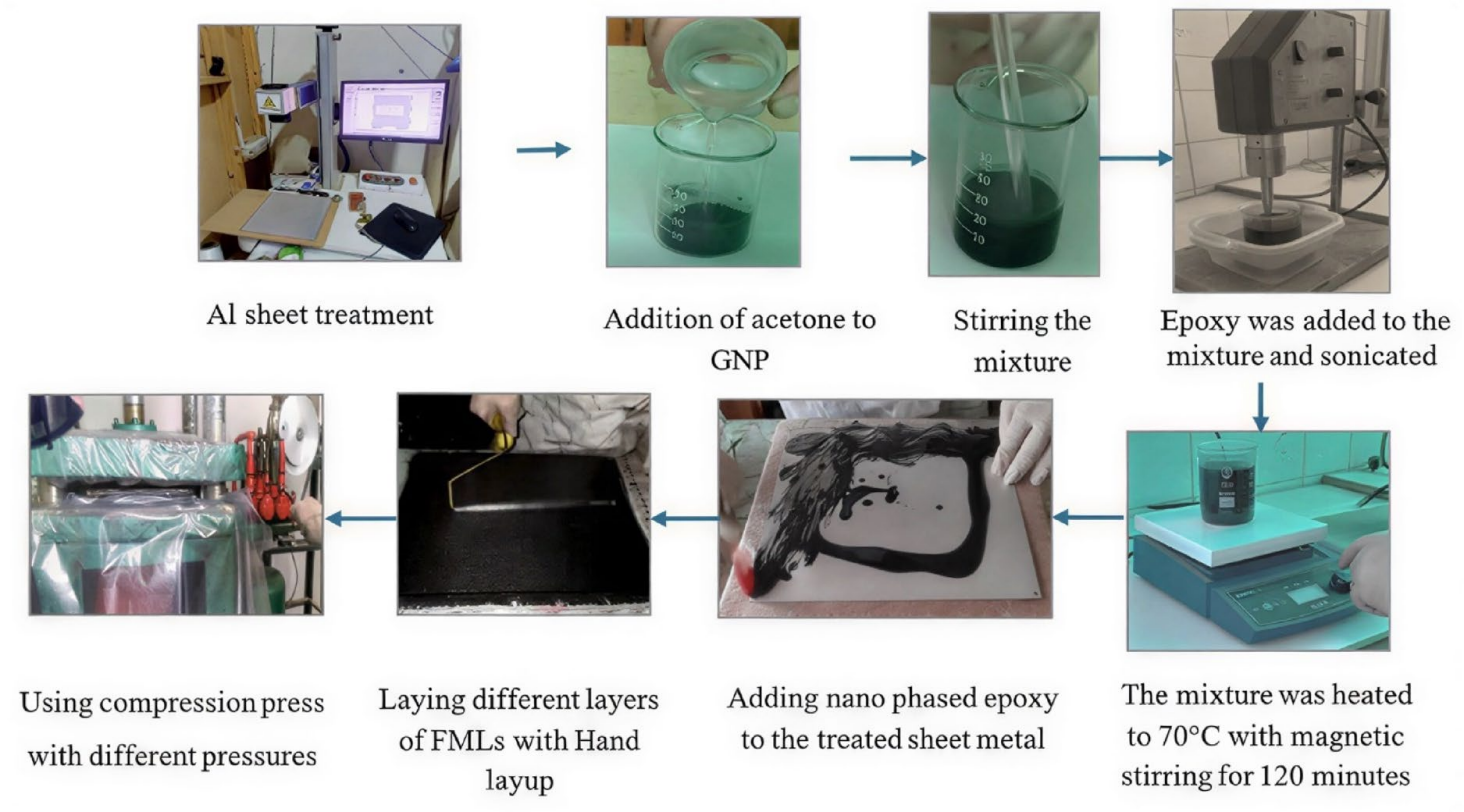
The steps of FMLs specimen manufacturing.

**Fig. 4:**
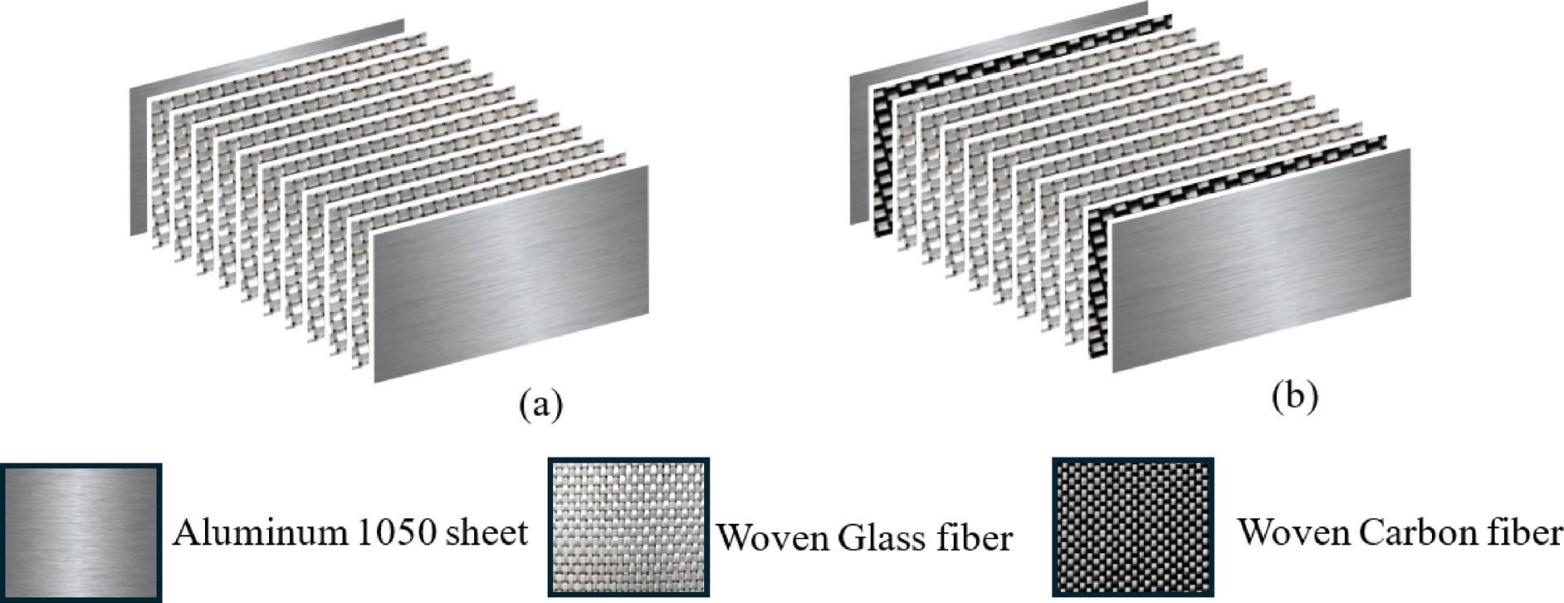
Schematic diagram of FMLs configuration (**a**) glass fiber reinforcement FMLs and (**b**) hybrid glass-carbon fiber reinforcement FMLs.

The steps involved in fabricating FMLs with nanocomposites in the core were depicted in Fig. 3. The acetone (10 weight% of epoxy’s weight) was added on a preset weight of nanofiller (0.1or 0.25 wt%). The Hielscher ultrasonic processor UP 200 S was used to blend the acetone/GNP combination for 120 min at a frequency of 24 kHz with a cycle time of 0.5 s. The mixture was placed in an ice water bath to cool it through the sonication process. After dissolving epoxy resin into the mixture, it was mixed for an additional 120 min while maintaining the same conditions. The resulting liquid was heated to 70 °C with magnetic stirring for 120 min at 250 rpm to evaporate the acetone from the mixture. After that, the viscous GNP and epoxy mixture was cooled to 30 °C. After adding the hardener gradually, the liquid was stirred for five minutes at 250 rpm using a magnetic stirrer. These processes improved the nanoparticles’ compatibility with epoxy resin, improved their dispersion, and broke up agglomeration. The FMLs were created using the hand-layup method, which was followed by compression molding under various pressures. After lay-up, the laminates were subjected to curing at room temperature for 24 h under constant consolidation pressures of 2, 5, and 7 bar, depending on the experimental condition. Following the 24 h curing period, the laminates were demolded and prepared for subsequent testing. No additional post-curing treatment was performed, as the objective of this study was to evaluate the influence of curing pressure under ambient-temperature conditions. Figure 4 shows a schematic diagram of FMLs configuration for both glass fiber reinforcement FMLs and hybrid glass-carbon fiber reinforcement FMLs. The glass fiber volume fraction of Glare is 39%. For hybrid FMLs the total fiber volume fraction was kept constant at 39%, comprising 31.5% glass fiber and 7.5% carbon fibers. Ten layers of fiber reinforcement are recommended to give a suitable thickness for the intended applications^13–15,41^.

### Tensile test

**Fig. 5:**
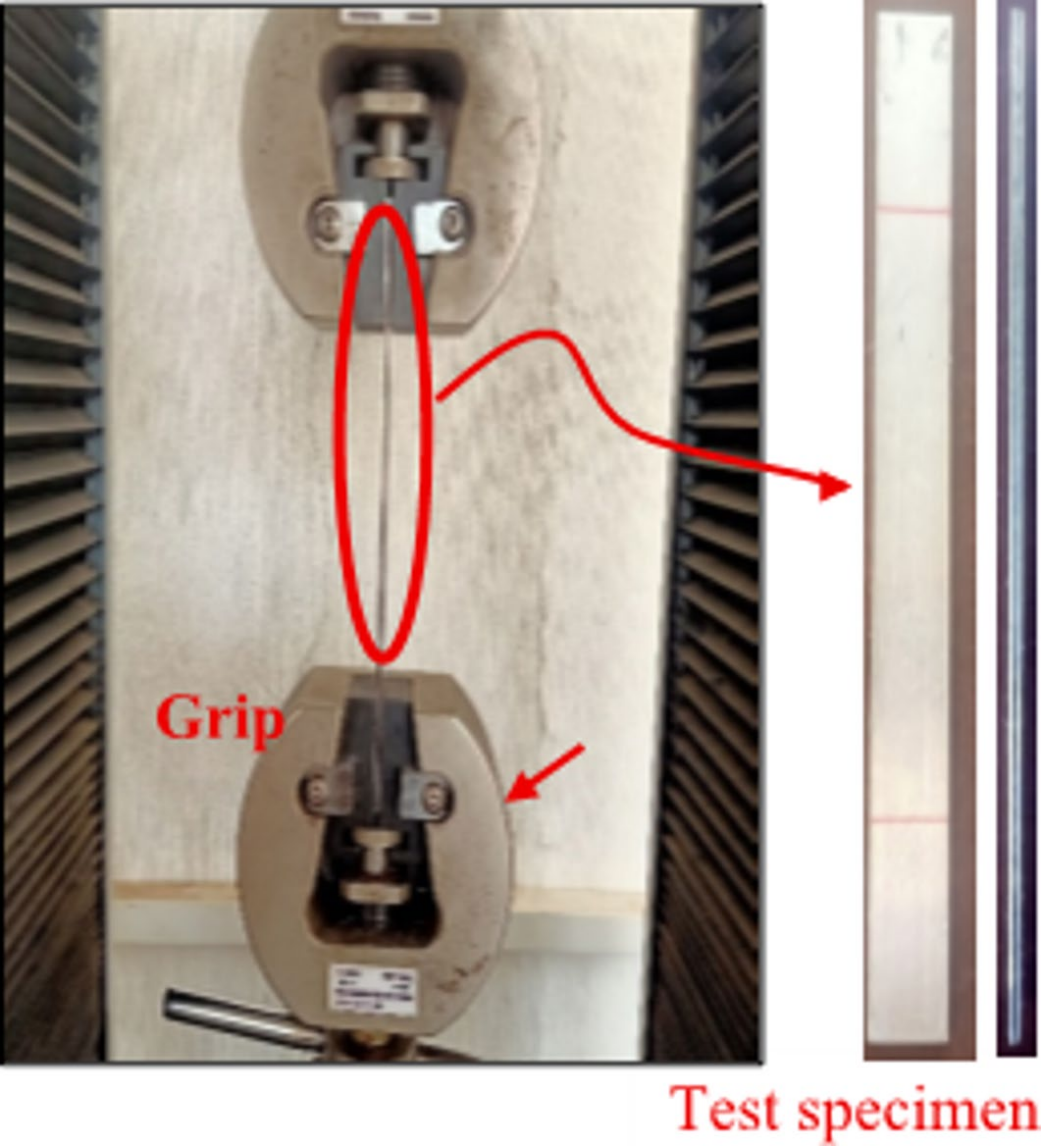
Tensile test setup for FMLs specimen.

According to ASTM D3039 tensile tests were performed using a Jinan Test Machine WDW 100 kN universal testing machine, Fig. 5, with a crosshead speed of 2 mm/min at room temperature (23 °C). The test samples were divided into strips that were 25 mm wide and 250 mm long. Five specimens of each variety of FMLs underwent tensile testing, and the average result was displayed. Carefully placing the specimens along the loading axis prior to the application of load ensured proper specimen alignment throughout tensile testing. To reduce eccentric loading and bending effects, the specimens were symmetrically secured using calibrated grips. Prior to every test, alignment was also visually confirmed to guarantee consistent load distribution and enhance the precision and reproducibility of the mechanical property measurements. The stress-strain curve acquired from the universal testing machine was used to compute the specimens’ tensile characteristics, including ultimate tensile strength, strain to failure, Young’s modulus, and toughness of the manufactured FMLs.

## Results and discussion

### Tensile engineering stress‑engineering strain curves

**Fig. 6:**
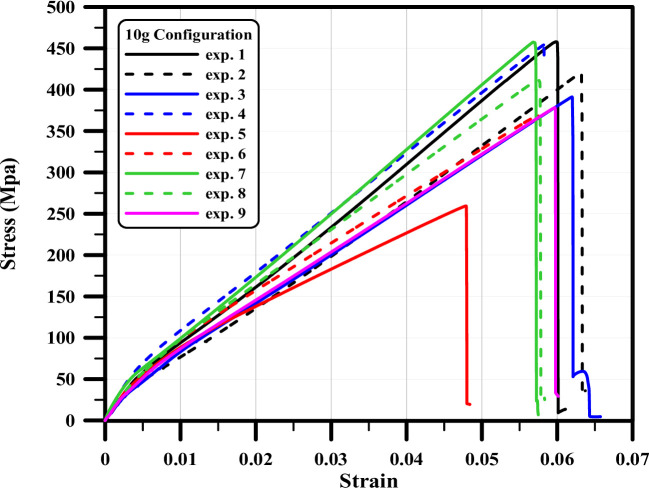
The stress–strain curves for 10 glass FMLs specimens.

**Fig. 7:**
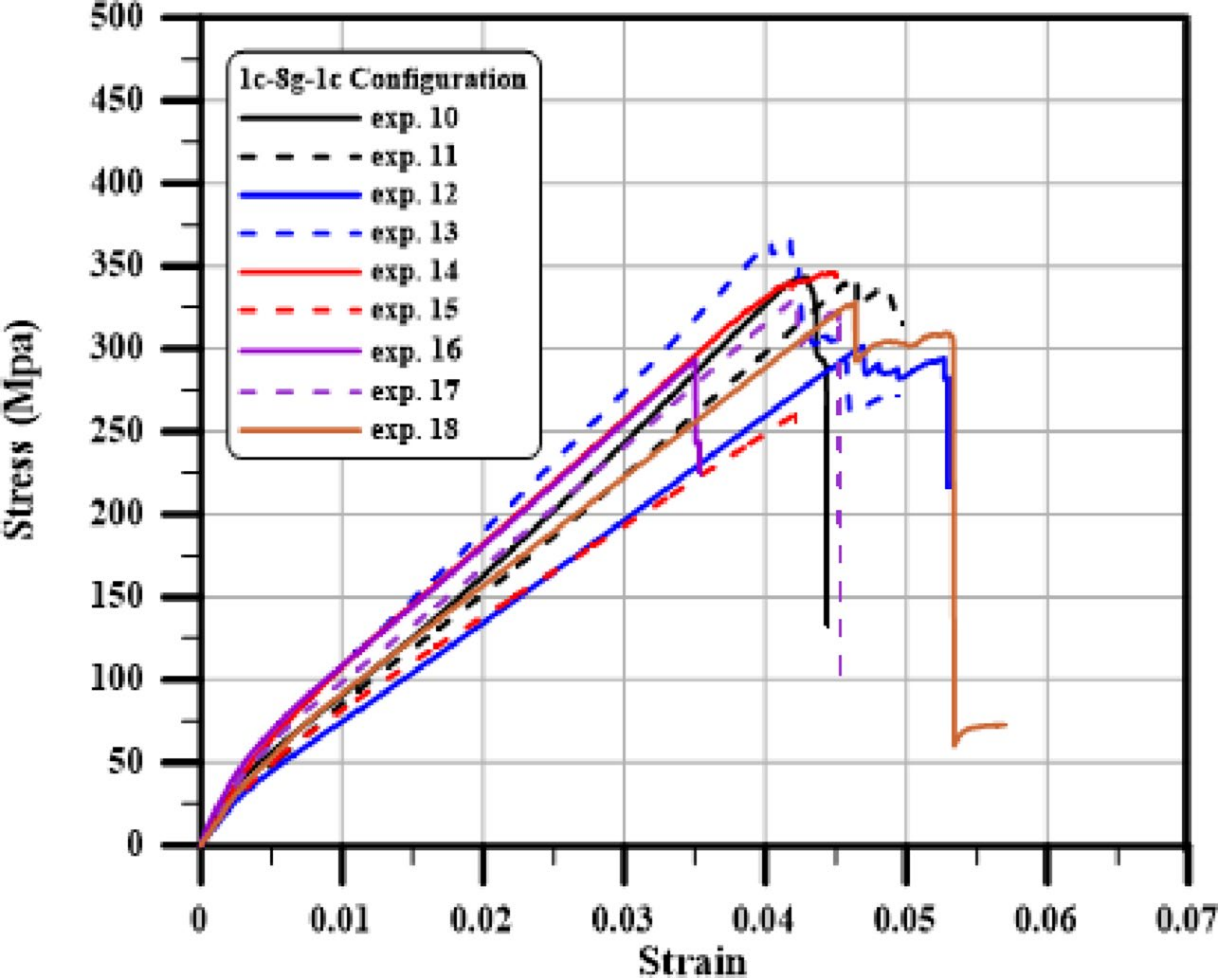
The Stress-strain curves for carbon-glass FMLs specimens.

Fiber metal laminates (FMLs) exhibit complex stress-strain relationships in the tensile loading mechanical behavior that illustrates the hybrid characteristics of these advanced composite materials. The stress-strain curves of the FMLs found in Figs. 6 and 7 exhibit distinct behaviors that indicate the sequential failure mechanisms of the hybrid structures. Understanding the response of distinct deformation regions and failure mechanisms are critical to optimizing the manufacturing parameters.

#### Tensile engineering stress‑engineering strain curves 10 glass FMLs specimens

The stress-strain behavior of the glass fiber laminates, Fig. 6, can be subdivided into three characteristic portions: the linear elastic region, the damage initiation region, and the failure region. For the initial linear elastic portion of the curve which extends from zero strain to approximately 0.01–0.02 strain. Stress–strain behavior is primarily dictated by the elastic deformation of the aluminum sheets and the glass fibers, while no significant micro-structural damage has occurred. This portion has a linear proportionality of the stress and strain consistent with Hookean behavior. The slope of this elastic portion reflects the effective modulus of elasticity of the glass fiber laminate composite which was in the 16–18 GPa range as summarized in Table 4. This elastic behavior was consistent with the findings of studies using GLARE-type laminates with respect to the effect of damage on the initial modulus which reflects the effective stiffness of the aluminum and the glass in the composite material prior to damage^42^.

The transition from linear to non-linear behavior represents the beginning of the second deformation region, defined from about 0.01–0.02 strain to 0.04–0.06 strain. Within this region, the stress–strain curves show progressive plastic deformation mechanisms resulting in a gradual decrease in slope. The non-linear behavior of the material occurs because of several concurrent mechanisms, including the beginning of plastic yielding in the aluminum layers, microcracking in the epoxy matrix, and progressive stress redistribution among the metal and composite phases. The variation in the degree of nonlinearity observed among the test specimens reflects the influence of parameters such as aluminum surface treatment, nanoparticle content, and processing pressure on the interfacial adhesion between layers as well as the specific mechanical response.

The final part of the curve, the failure region, is characterized by a rapid drop through a maximum stress (in the range of 370–470 MPa depending on the sample) at a strain of 0.04–0.06. The fracture morphology, Fig. 6, shows that the failure region is associated with progressive failure in which energy is dissipated via fiber pull-out, fiber breakage and delamination (layered). This type of progressive failure is typical of glass-based FMLs and suggests better damage-tolerance and toughness. There was also matrix cracking and delamination at the aluminum, glass composite interface.

#### Tensile engineering stress‑engineering strain curves for carbon-glass FMLs specimens

The stress strain curves of the hybrid glass carbon laminates shown in Fig. 7 show significantly different behaviors and show individual parts for elasticity, damage initiation and failure. The first linear elastic portion of the curve, from zero strain to about 0.01–0.02 strain, had a steeper slope compared to the glass fiber metal laminates (FMLs). The steeper slope indicates that the modulus of elasticity is higher which means that the stiffness is also much higher. This change is due to carbon fibers, which are significantly stiffer than glass fibers.

As the load continues to progressively increase beyond the elastic limit, the damage initiation region is clearly marked by the initial appearance of interfacial cracks between the fiber types. The hybrid structures create a poorly matched stiffness within the stiffness of the carbon and glass plies. The resultant layers create interlaminar shear stresses that induce delamination between the two layers at a particularly low strain. This behavior is observed with a greater magnitude in the early portion of the transition away from linearity in Fig. 6 compared to Fig. 7. Similar observations of these phenomena have been noted in hybrid FMLs studies where it was determined that carbon inclusions provided improved stiffness properties, however, they also resulted in earlier delamination and matrix cracking^43^. The stress concentration at the interface promotes the transition from minor damage to catastrophic failure.

The failure zone of the hybrid laminates exhibits a rapid stress reduction following the peak load, characterized by minimal plasticity or progressive failure. Figure 6 illustrates the fracture morphology, revealing notable delamination between the aluminum and composite layers, as well as between the carbon and glass fiber layers. This observation indicates that the abrupt decrease in load-bearing capacity is attributable to interfacial instability. In contrast to glass-based laminates, which exhibit fiber pull-out and a progressive failure mode, carbon-glass hybrids display a brittle fracture characterized by carbon fiber breakage and interface separation. This observation aligns with contemporary research indicating that carbon-containing fiber metal laminates experience a decrease in toughness and energy absorption in favor of increased stiffness and strength^44^. The failure strain ranged from 0.035 to 0.064 and the ultimate tensile strength varied from 254.15 to 472.12 MPa, indicating an increase in ductility of about 82.9% between the laminates with the lowest and greatest performance. Similarly, the toughness improved by almost 171.2%, from 5.63 to 15.27 MPa, suggesting a far higher capability for energy absorption before failure. Wider deformation regions were typically associated with higher failure strain and toughness values in the specimens, indicating that damage accumulated gradually prior to final breakage. On the other hand, specimens with smaller deformation regions showed poorer toughness and strain-to-failure, suggesting earlier crack initiation and faster crack propagation.

### Mechanical properties of FMLs for each experiment

The tensile properties obtained for manufactured fiber metal laminates (FMLs) exhibited a clear dependence on configuration, surface treatment and hybridization with carbon fiber. The tensile properties are shown in Table 4. ultimate tensile strength of approximately 472 MPa, specimen 1, was achieved with glass laminates and chemically treated aluminum, while the minimum ultimate tensile strength of approximately 254 MPa, specimen 5, obtained with glass laminates and laser pattern X1 treated. The improved performance of sample1 could be associated with good fiber-metal adhesion, where the surface treatment of the aluminum minimized premature delamination and facilitated stress transfer. According to the findings, chemical bonding was the primary adhesion mechanism in this investigation, with mechanical interlocking coming in second. When compared to the laser-treated aluminum specimens, the chemically treated specimens continuously demonstrated higher tensile strength, hardness, and failure strain. Chemical treatment removes contaminants and weak surface layers thus promotes adhesion by strengthening interfacial bonding and making the aluminum surface more wettable toward the epoxy matrix, while laser texturing mainly increases adhesion by mechanical interlocking.

Similar results have been reported in studies where the surface of aluminum was chemically treated or laser treated, increasing the load bearing capability of GLARE-type laminates^45^. The lower strength of some glass-carbon hybrid laminates, such as sample 15, could be attributed to the interfacial incompatibility between the carbon and glass laminates, with the carbon fiber causing an additional delamination failure mechanism under tensile loading and leading to premature fracture^46^.

The failure strain values of the specimens (between 0.035 and 0.065) show the low ductility of FMLs relative to monolithic metals. While glass carbon hybrids had lower ductility (as evidenced by the minimum of 0.035 for sample 16), glass-based laminates typically showed a higher strain of failure - seen in sample 3 with a strain to failure of 0.065. The decreased strain capacity of carbon-containing laminates denotes the brittle nature of carbon fibers and their tendency to concentrate on stress at the interface, hence speeding cracking. Recent tests of hybrid laminates showed comparable results; adding carbon fibers increased stiffness and decreased breaking elongation^47^.

The tensile modulus for the laminates was between 7 and 17 GPa. Some of the hybrid laminates had relatively high stiffness because of the use of high-modulus carbon fibers. However, stiffness did not correspond with strength; Glare had better ultimate stresses and strains than the carbon-reinforced FMLs. This indicates that the carbon fibers, in the proportions and lay-up used here, increased the rigidity of the laminates, but decreased ductility without improving the peak load capacity^48^. This is likely due to lower strain-to-failure of carbon fibers and the possibility of premature fiber breakage or interfacial stress concentration in the hybrid lay-ups.

The toughness modulus values, which show the energy absorbed up to fracture, ranged greatly from 5.6 MPa for sample 16 to 15.3 MPa for sample 1. Glass-based laminates registered greater toughness values; in particular, sample 1 showed more tensile strength and energy absorption characteristics. Because of energy dissipation via fiber pullout and matrix cracking, this result implies associated glass fiber arrangements were better in progressive failure. The extremely low toughness values associated with the carbon-glass laminate samples (e.g., specimens 15 and 17) showed that carbon fiber fracture characteristics related to catastrophic fracture behavior. GLARE-type laminates have demonstrated better damage tolerance and energy absorption than hybrid carbon-glass ones, therefore supporting their possible uses in situations needing impact resistance^17^.

**Table 4:** Mechanical properties of FMLs for each experiment.

Experiment	Ultimate tensile strength (MPa)	Tensile modulus (GPa)	Toughness (MPa)	Failure strain
1	472.12 ± 29.21	12.99 ± 1.39	15.27 ± 0.83	0.064 ± 0.0025
2	401.74 ± 36.7	11.85 ± 0.07	12.42 ± 0.16	0.060 ± 0.0055
3	386.19 ± 10.39	10.69 ± 0.55	12.93 ± 0.14	0.065 ± 0.0088
4	459.37 ± 7.63	17.45 ± 0.103	14.88 ± 1.46	0.059 ± 0.0031
5	254.15 ± 12.87	12.75 ± 1.04	6.96 ± 0.428	0.047 ± 0.0009
6	360.16 ± 16.60	12.77 ± 0.67	11.31 ± 1.211	0.057 ± 0.0037
7	438.99 ± 32.86	15.65 ± 0.97	12.74 ± 1.49	0.056 ± 0.0027
8	410.68 ± 30.59	14.05 ± 0.62	12.50 ± 1.52	0.057 ± 0.0028
9	365.34 ± 18.08	11.88 ± 0.95	11.86 ± 1.38	0.060 ± 0.0041
10	374.01 ± 28.08	7.72 ± 0.17	8.73 ± 0.88	0.045 ± 0.0014
11	339.31 ± 14.28	11.75 ± 0.88	8.66 ± 1.18	0.047 ± 0.0037
12	299.32 ± 7.71	10.44 ± 0.18	8.53 ± 0.92	0.052 ± 0.0008
13	370.45 ± 5.52	15.86 ± 0.85	9.18 ± 1.13	0.045 ± 0.0037
14	344.56 ± 16.49	15.16 ± 1.49	8.38 ± 0.58	0.044 ± 0.0012
15	256.00 ± 4.34	13.11 ± 0.9	5.68 ± 0.29	0.040 ± 0.0018
16	288.42 ± 23.20	16.33 ± 1.02	5.63 ± 0.78	0.035 ± 0.0017
17	328.96 ± 25.52	14.05 ± 0.59	8.53 ± 0.60	0.047 ± 0.0013
18	319.81 ± 6.75	11.59 ± 0.77	9.27 ± 1.05	0.051 ± 0.0022

### Failure mechanisms

**Fig. 8:**
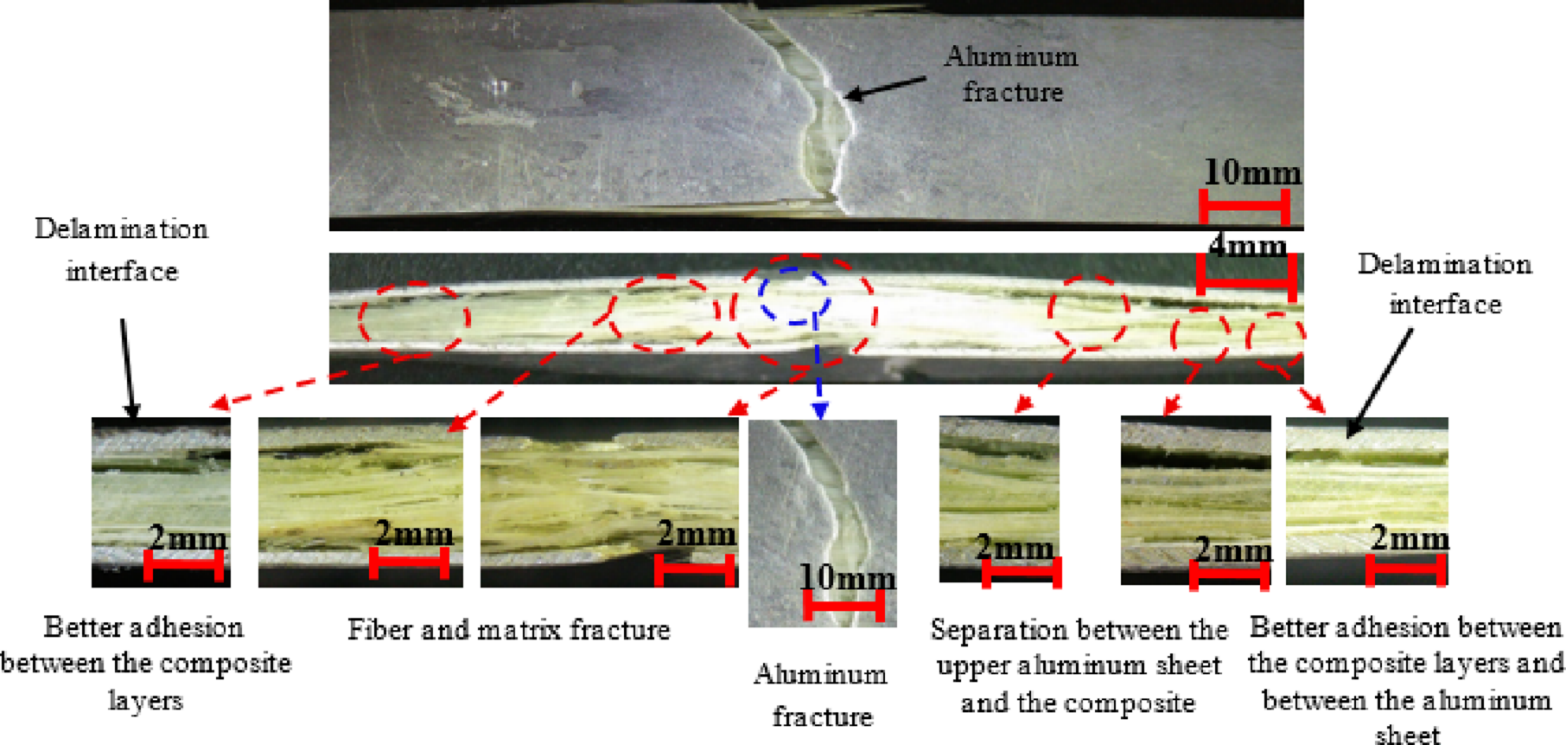
Optical micrographs of the fracture morphology (experiment 1).

**Fig. 9:**
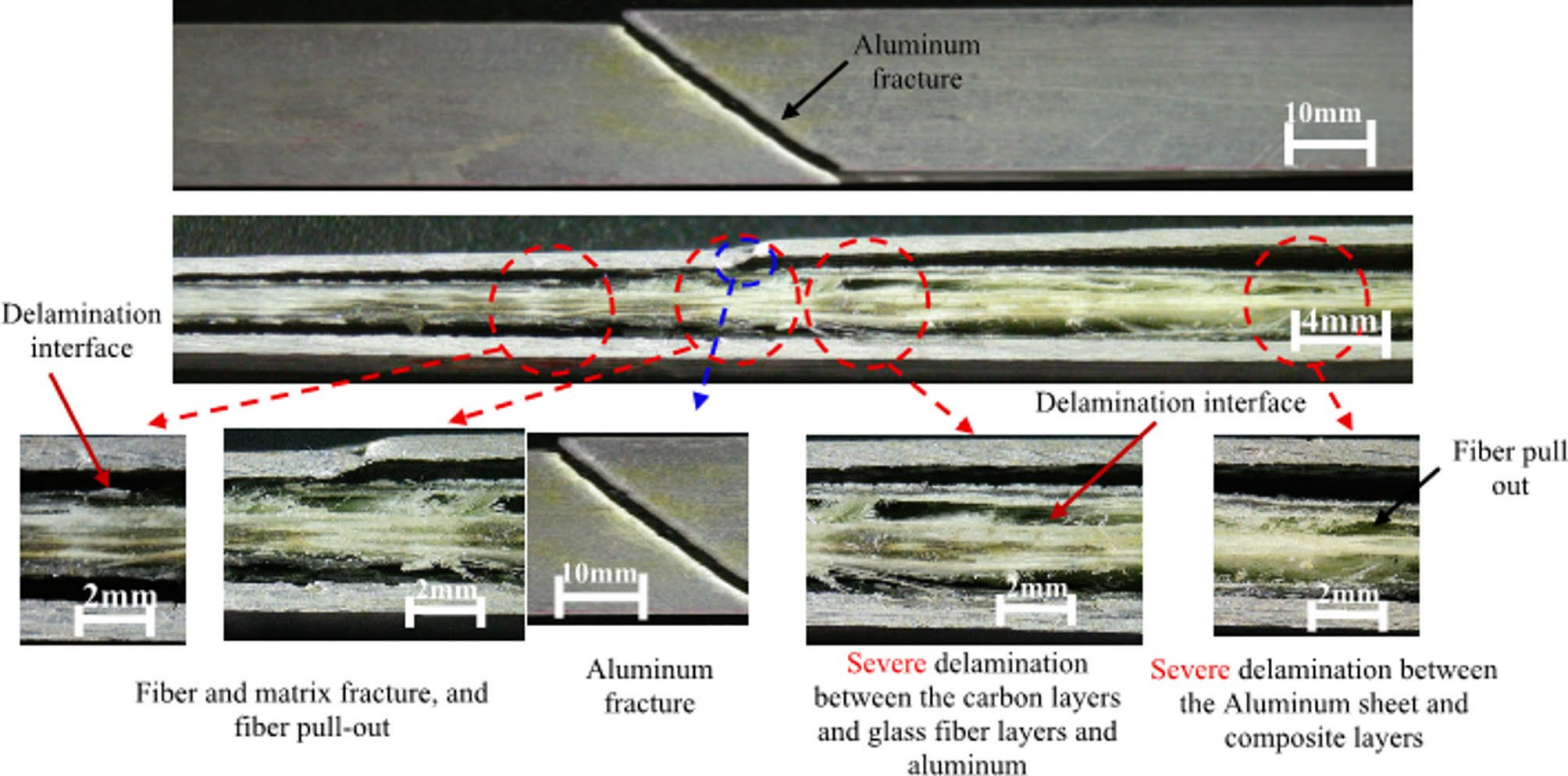
Optical micrographs of the fracture morphology (experiment 15).

Figures 8 and 9 display the images of the tensile fracture mechanism of experiments 1 and 15 respectively. The two specimens had the highest and lowest tensile strength, respectively. These two samples were compared in terms of their fracture mode to comprehend the whole range of failure processes. The fracture morphology of specimen 1 exhibited one of the best mechanical properties in terms of toughness of 15.27 MPa, the highest tensile strength of 472.12 MPa, and a large strain of 0.064 (Table 4). It displayed a complex fracture morphology that can elucidate some of the failure modes operating within high-performance glass fiber metal laminates. The optical micrographs in Fig. 8 indicate several failure modes that were occurring simultaneously, across several length scales, with similar fracture modes that indicate the hybrid nature of the laminated structure; and the excellent interfacial adhesion achieved through the chemical surface treatment used in this specimen. The most significant feature noted in the thickness direction of the fracture surface was better adhesion between the composite layers. This finding is directly linked to the exceptional mechanical performance exhibited by the specimen, where the treatment of the chemical surface treatment resulted in enhanced interfacial bonding between the aluminum substrate and glass fiber-epoxy composite layers. Recent research has highlighted that, for fiber-reinforced polymer (FRP) and fiber metal laminate composites, interlaminar fracture (or delamination) is one of the significant failure modes; thus, achieving greater interfacial adhesion between the layers is imperative to maximize mechanical performance. For specimen 01, the strong adhesion observed is evident because this specimen is at the top of the stress-strain curve (Fig. 4) and shows the highest ultimate strength of all the specimens tested.

The micrographs indicate that the presence of “aluminum fracture” demonstrates efficient stress transfer from composite to metal. As the mechanism of failure observed indicates that the interface strength was greater than the ultimate tensile strength of the aluminum, it is believed to reflect the best adhesion possible [50]. In addition, the failure mode indicates that load transfer between the aluminum and composite constituents was effectively maintained during deformation by the individual constituents, thus allowing both materials to contribute to the maximum load carrying capacity. This also implies significant amount of plastic deformation in the aluminum layers occurred before final fracture, which helps to explain the exceptional toughness value of 15.27 MPa that was recorded for this specimen.

The composite regions showed “fiber and matrix fracture, and fiber pull-out” according to the micrograph’s investigation. These mixed failure mechanisms are characteristic of well-bonded fiber-reinforced composites, and the interfacial bonding is sufficient for the fibers to fracture rather than merely deboned. Previous studies indicate that fiber pullout is a progression from initial debonding at the fiber-matrix interface to crack propagation to complete pullout of the fiber under load. The presence of both fiber fracture and fiber pull-out means there is an optimum ratio of interfacial bonding, in that some fibers failed in tension while others were pulled out from the matrix, which provides an effective mechanism for dissipating energy, contributing to the high toughness of this specimen. The slight crack and separation between the upper aluminum sheet and the composite observed in certain regions represents localized delamination events that occur during the final stages of failure. Despite this localized separation, the overall integrity of the interface is maintained across most of the fracture surface, as evidenced by the exceptional mechanical properties achieved. This behavior is consistent with the stress-strain response shown in Fig. 6, where specimen 1 demonstrates a relatively smooth curve without significant load drops that would indicate catastrophic delamination events. The presence of minor interfacial cracking without complete delamination suggests that the chemical surface treatment was effective in creating a durable bond that could accommodate significant deformation before failure.

The micrographs of specimen 15 (a glass-carbon hybrid system (1c + 8 g + 1c)) show a different failure character than observed in the pure glass fiber system. Specimen 15’s mechanical properties were some of the lowest, including a tensile strength of 256.00 MPa, a toughness of 5.68 MPa, and a failure strain of 0.040 (Table 4). The amount of damage noted under optical microscopy (Fig. 8) helps to explain this very low mechanical performance and conveys some of the difficulty of producing proper interfacial compatibility in any hybrid fiber system.

The significant feature observed in the fracture morphology is the severe delamination between the aluminum sheet and composite layers, which is a damaging failure mode that greatly reduces the load-carrying capacity of the laminated structure. This substantial delamination directly elucidates the reduced tensile strength, and toughness values for specimen 15 when compared to the high-performance glass fiber specimens. Recently, it has been established that delamination is one of the most detrimental failure modes in composite laminates since it inhibits load transfer between the composite laminate layers and causes failure of the laminate at stress levels that are many times lower than the individual materials capacity. The extent of the delamination in specimen 15 corresponds to its location at the lower scaling of the stress-strain curves shown in Fig. 7, where hybrid specimens tended to show poorer performance than pure glass fiber systems. Laser treatment is the method used to enhance adhesion, but its effectiveness largely depends on the specific parameters used (such as power, speed). Improperly optimized laser treatment can lead to the creation of a heat-affected area or a surface unsuitable for strong bonding with epoxy resin. In this case, it seems that laser treatment was less effective than chemical treatment for sample 1 in enhancing strong adhesion. The higher curing pressure of 5 bar used for this sample would generally be expected to improve consolidation and reduce voids, which should theoretically enhance interfacial strength. However, the observed severe delamination suggests that the negative impacts of the hybrid fiber configuration and potentially suboptimal laser surface treatment overshadowed any benefits from the increased pressure. The higher pressure might have also induced higher thermal residual stress during cooling, which can be detrimental to interfacial integrity, especially in a hybrid system with materials of varying thermal expansion. Nanoparticles (0.25%) could also agglomerate at the interface, acting as stress concentrators instead of reinforcements.

The observation of severe delamination between the carbon layers and glass fiber layers indicates an interfacial compatibility issue that is unique to hybrid fiber systems. The hybrid configuration, which includes both glass and carbon fibers, results in a stiffness mismatch between the two layers. Carbon fibers, characterized by their high modulus, restrict deformation, whereas the glass fibers, with their lower modulus, experience larger strains. This difference generates shear stresses at the interface of the two plies. Such a mismatch contributes to the delamination observed^51^. The presence of aluminum fracture in the specimen indicates that despite the extensive delamination, the aluminum layers still contributed to load carrying until their ultimate failure. However, the reduced effectiveness of load transfer due to delamination means that the aluminum layers could not reach their full potential, contributing to the overall reduced mechanical properties. The composite regions exhibit “fiber and matrix fracture, and fiber pull-out” like the glass fiber specimen, but the effectiveness of these energy dissipation mechanisms is severely compromised by the extensive delamination observed throughout the fracture surface. The interaction between carbon and glass fibers appears to have created interfacial weak points that facilitated crack initiation and propagation, leading to the premature failure observed in this specimen.

**Fig. 10:**
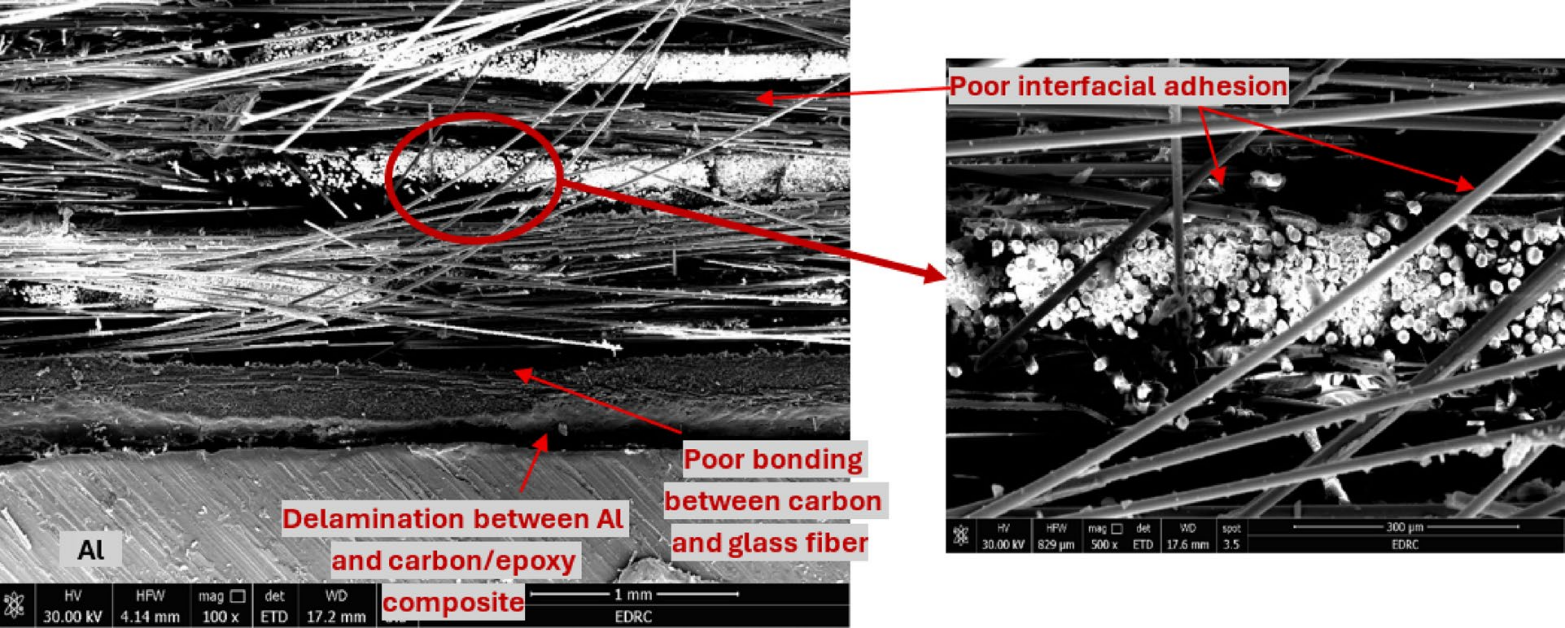
SEM images for fracture morphology of hybrid carbon/glass reinforced FMLs (experiment 17).

The hybrid carbon/glass FML (Experiment 17) showed heterogeneous failure characteristics in the SEM micrographs as shown in Fig. 10. The glass-reinforced areas were able to successfully support load transfer because good bonding was seen between the glass fiber plies and between the matrix and glass fibers. However, there was also noticeable poor adhesion between the carbon and glass fiber layers, as well as localized delamination between the aluminum sheet and the carbon/epoxy composite. Under tensile loading, this weak interfacial adhesion enhanced crack propagation and encouraged break initiation. Thus, the ultimate tensile strength of Experiment 17 is 328.96 MPa and toughness of 8.53 MPa which with 30.3% and 44.1% lower, respectively, than those of the ideal specimen. The ideal specimen is Experiment 1 which has ultimate tensile strength of 472.12 MPa and toughness of 15.27 MPa. Additionally, the failure strain dropped by around 26.6%, from 0.064 to 0.047. These findings suggest that the primary failure mechanism governing the hybrid laminate’s tensile response was interfacial delamination rather than fiber breakage. Strength and energy absorption capacity were limited as a result of the interlaminar shear stresses created by the observed stiffness mismatch between carbon and glass plies, which weakened the interface and decreased the effectiveness of stress transmission.

**Fig. 11:**
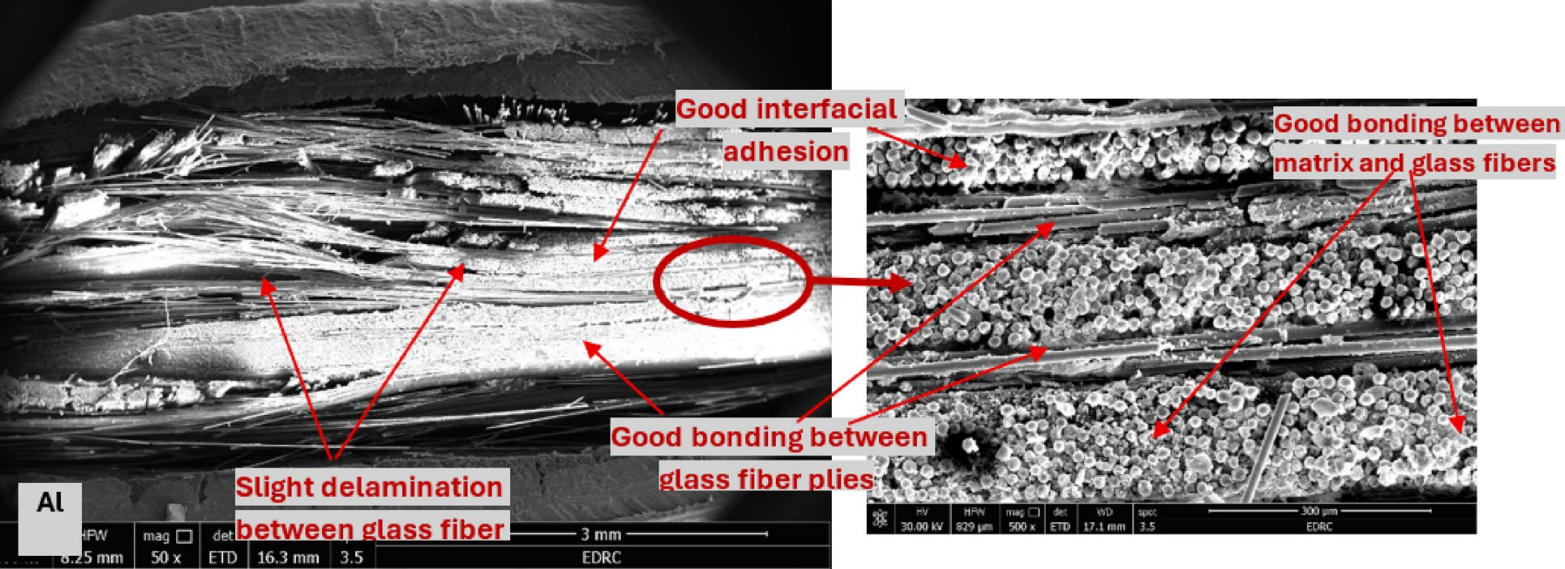
SEM images for fracture morphology of glass reinforced FMLs (experiment 5).

Compared to the hybrid arrangement, the glass-reinforced metal laminate (Experiment 5) showed a more consistent fracture morphology with fewer interfacial incompatibilities. The lack of carbon/glass interfacial mismatch decreased the probability for severe delamination, even if Experiment 5 did not attain the best mechanical characteristics. Tensile strength and toughness values of 254.15 MPa and 6.96 MPa, respectively, indicate that matrix cracking, fiber fracture, and localized interfacial debonding, rather than catastrophic interlaminar separation, were the main causes of failure as shown in Fig. 11. The toughness was about 22.6% lower than in Experiment 17, suggesting that factors other than laminate configuration, such as surface treatment and processing conditions, also affected the fracture behavior. However, the SEM data verify that optimizing the tensile performance of FMLs structures requires preserving interfacial integrity.

**Fig. 12:**
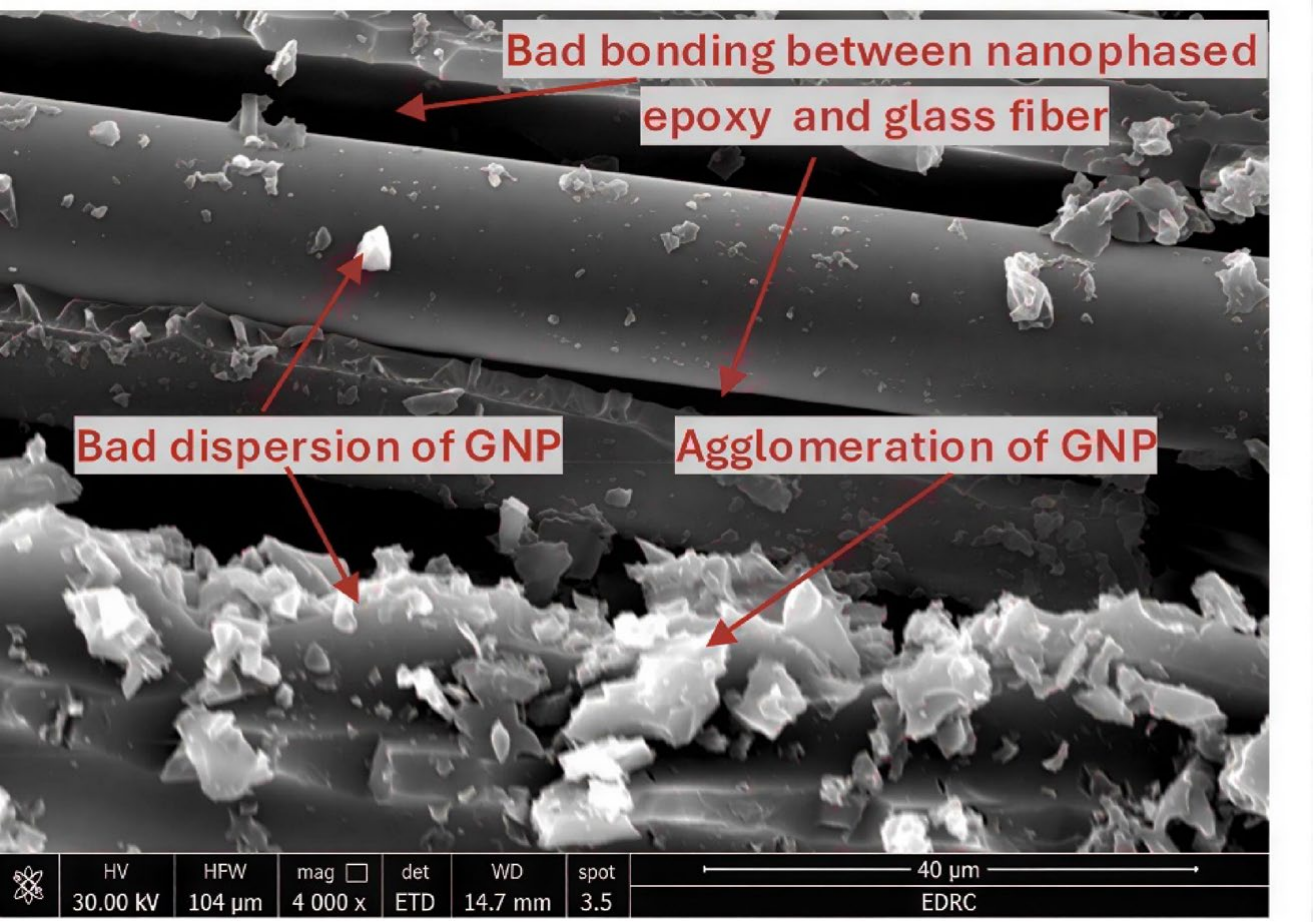
SEM image for the fracture morphology of FMLs containing 0.1% GNP (experiment 2).

**Fig. 13:**
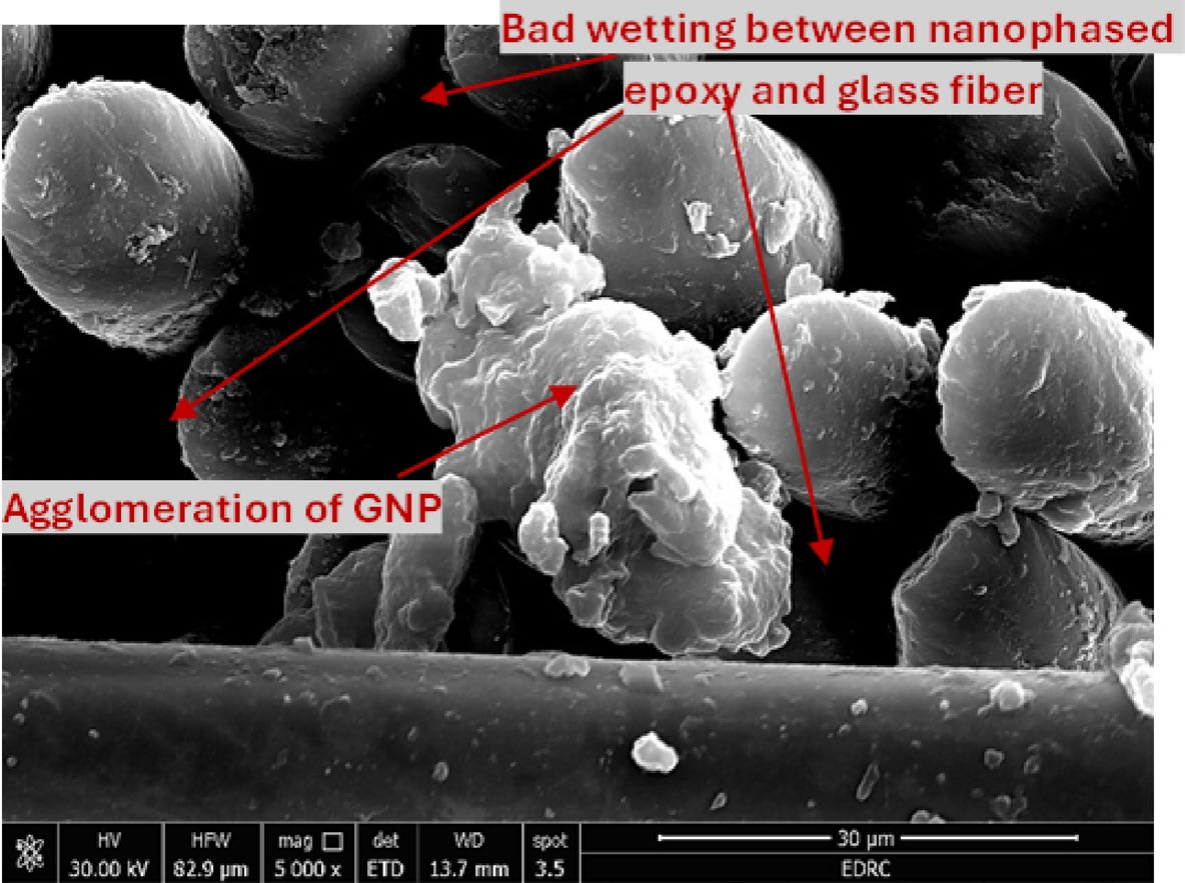
SEM image for the fracture morphology of FMLs containing 0.25% GNP (experiment 16).

Significant nanoparticle aggregation and uneven dispersion within the epoxy matrix were visible in the SEM pictures of the graphene-modified FMLs. Figure 12 shows localized clusters of graphene nanoplatelets and weak adhesion between the nanophase epoxy and glass fibers were noted for Experiment 2 with 0.1 weight% GNP. These agglomerations serve as locations of stress concentration, which promotes the development of microcracks and lessens the efficiency of load transfer across the fiber-matrix interface. Agglomerates prevented the nanoparticles from reaching their maximum reinforcing potential, even though Experiment 2 produced a tensile strength of 401.74 MPa. Despite the addition of graphene reinforcement, the tensile strength was still about 14.9% below the maximum value found in Experiment 1. A similar behavior was observed for Experiment 16 containing 0.25 wt% GNP, where pronounced agglomeration and poor wetting between the nanophased epoxy and glass fibers were detected as shown in Fig. 13. These flaws had serious mechanical effects. When compared to the ideal laminate, Experiment 16’s tensile strength of 288.42 MPa, toughness of 5.63 MPa, and failure strain of 0.035 showed reductions of 38.0%, 63.1%, and 45.3%, respectively. The sharp decline in performance suggests that raising the graphene content above the effective dispersion limit encourages particle clustering, which leads to localized stress concentrations and accelerates the development of cracks. With delta values of just 22.8 MPa for tensile strength and 0.97 MPa for toughness, these findings explain why nanoparticle content had the lowest statistical contribution to tensile properties in the Taguchi study. Consequently, rather than graphene addition alone, laminate configuration and interfacial adhesion quality were the main factors influencing the mechanical performance of the studied FMLs.

Overall, the SEM findings show that tensile performance and interfacial integrity are directly correlated. Higher tensile strength, toughness, and ductility were demonstrated by specimens with good bonding and little delamination, while laminates with interfacial flaws, fiber incompatibility, and nanoparticle aggregation failed earlier and had less capacity to absorb energy. These results corroborate the ANOVA findings, which showed that surface treatment and laminate configuration were the main variables influencing the tensile behavior of the manufactured FMLs.

### Taguchi analysis

**Fig. 14:**
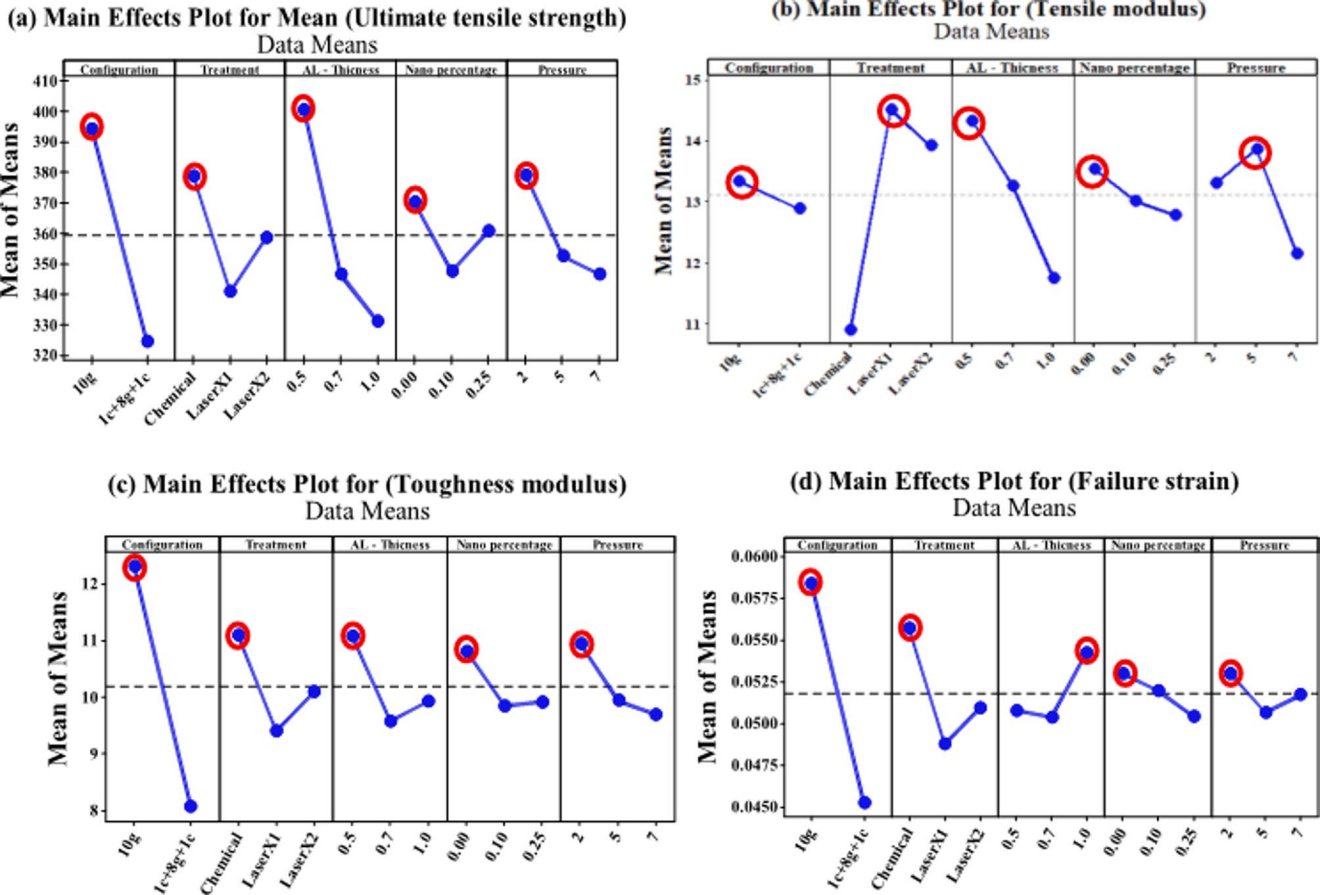
The main effect graphs for (**a**), Ultimate tensile strength (**b**), Tensile modulus, (**c**) Toughness modulus, and (**d**) Failure strain.

To analyze the experimental results, MINITAB software was utilized to perform the Taguchi analysis. The main effect and S/N ratio were derived to evaluate the influence of the manufacturing factors shown in Table 1 on the resultant FMLs.

mechanical properties shown in Table 4. A validation experiment was then conducted for the comparison between predicted and experimental values of tensile strength. The average values for ultimate tensile strength, tensile modulus, toughness modulus and failure strain for each variable at each level were assessed and presented in Table 5. The main effect graphs for these properties, illustrated in Fig. 14, were generated. In the main effect plots, deviations from the horizontal line indicate that the process parameter has a greater impact on the response parameters. The quality characteristics evaluated in this experiment follow the “larger-is-better” principle for FML’s mechanical properties. The S/N ratio for “larger-is-better” can be calculated by Eq. (1).

1 $${\rm{S}}/{\rm{N }} = {\rm{ }} - {\rm{1}}0{\rm{ }} \times {\rm{ lo}}{{\rm{g}}_{10}}{\rm{ }}\left[ {\left( {{\rm{1}}/{\rm{n}}} \right){\rm{ }} \times {\rm{ }}\Sigma \left( {{\rm{1}}/{{\rm{y}}^2}_i} \right)} \right]$$

where ‘‘n’’ is the number of observations and y is the observed data.

### Properties analysis

The tensile strength analysis indicates that laminate configuration has the greatest influence, followed by aluminum thickness. This conclusion is shown with the steep slope for these two factors in Fig. 14a. The response table for means, Table 4, supports this, with the laminate configuration having the highest delta (69.8 MPa) and being ranked first, which means it has the most influence on this property. Following closely was aluminum thickness (delta = 69.4 MPa and rank 2), as both the layup sequence and the thickness of the metal sheet matter greatly when determining the ultimate strength of the laminate. The type of aluminum surface treatment (delta = 38.0 MPa and rank 3) and the applied pressure during curing (Factor E, delta = 32.6 MPa and rank 4) showed the same significant but comparatively lesser effect, and the percentage of nanoparticles (delta = 22.8 MPa and rank 5) showed the least effect on tensile strength. Using the peak values present in Fig. 14a, the optimum conditions to maximize tensile strength are to use the “10 g” configuration with chemical surface treatment with a thickness of aluminum of 0.5 mm, a nano percentage of 0.0%, and a curing pressure of 2 bar. Tretiak et al. [52] found that structural composites with a porosity criterion of less than 2% are usually acceptable and that the small void volume fraction does not affect tensile modulus. Liu et al. [53] showed that cure pressures as low as 0.2 MPa can reduce void content below 2% if the cure temperature and dwell time are controlled and the resin system has good flow. The 2-bar specimens had the highest tensile strength and modulus at all pressure levels, indicating that void content was acceptable for this material system.

The tensile modulus response, as shown in Table 5; Fig. 14b, has a different parametric hierarchy.

than tensile strength, with surface treatment as the most important (rank 1, delta = 3.61). Aluminum thickness was the second most important parameter that had an influence on the modulus of elasticity (delta = 2.59), while the pressure remained third in rank in their influence on Young’s modulus. In contrast, it would appear that the nano percentage and Configuration have little effect on the elastic modulus to achieve maximal stiffness. It is also noteworthy that, according to the peak values shown in Fig. 14b, the optimal parameters include a laser 1 mm scanning texture, glass fiber laminate configuration, aluminum thickness of 0.5 mm, 0% graphene nanoparticles, and a curing pressure of 5 bars.

The toughness modulus analysis in Table 5 shows that configuration remains the most influential (rank 1, delta = 4.25 MPa) encouraging a material selection perception in energy absorption behavior. The influence of toughness is ranked in this order: configuration, surface treatment (rank 2, delta = 1.69), aluminum thickness (rank 3, delta = 1.50), processing pressure (rank 4, delta = 1.24), and percentage of nanoparticles (rank 5, delta = 0.97). The ideal set of parameters to achieve the most toughness, observed in Fig. 14c, is ‘10 g’ configuration, chemical treatment, aluminum thickness of 0.5 mm, with 0% nanoparticles, and pressure set at 2 bar.

The failure strain analysis, as presented in Tables 5 and illustrated in Fig. 14d, reveals configuration as the most influential parameter (rank 1, delta = 0.013), followed by surface treatment (rank 2, delta = 0.007), aluminum thickness (rank 3, delta = 0.004), nanoparticle percentage (rank 4, delta = 0.003), and processing pressure (rank 5, delta = 0.002). According to Fig. 14d, the combination for achieving the highest ductility is a ‘10 g’ configuration, chemical treatment, an aluminum thickness of 1.0 mm, 0% nanoparticles, and a pressure of 2 bar.

#### Comparative parameter ranking analysis

The examination of the ranking in Table 5 shows that configuration consistently ranks as the most important parameter for three of the four mechanical properties (tensile strength, toughness, and failure stress), thus demonstrating its basic importance in FMLs design. As noted in both Table 5; Fig. 14b, Surface treatment also demonstrates variable importance; it ranks first in tensile modulus but second in toughness and failure strain, indicating that it plays a complex role in determining various mechanical properties. The thickness of aluminum also shows consistently moderate importance across all properties and confirms the critical importance of the proper thickness of aluminum in the manufacturing of these FMLs. The consistently low ranking of the nano percentage and pressure parameters across all mechanical properties suggests that these parameters, although they may be important for other properties such as fatigue resistance or environmental durability, are relatively unimportant for determining fundamental mechanical properties. This finding suggests that to improve FMLs, we should focus primarily on configuration selection, how thick the aluminum should be, and what surface treatment to use, not on the use of nano reinforcement or the change in pressure within the ranges examined.

**Table 5:** Response table for means tensile strength, tensile modulus, toughness modulus, and failure strain.

Tensile properties	Level	Configuration	Treatment	AL-thickness	Nano percentage	Pressure
Ultimate tensile strength (MPa)	1	394.3	378.8	400.6	370.2	379.2
	2	324.5	340.8	346.6	347.4	352.6
	3	–	358.7	331.1	360.7	346.5
	Delta	69.8	38.0	69.4	22.8	32.6
	Rank	1	3	2	5	4
Tensile modulus (GPa)	1	13.34	10.91	14.33	13.54	13.32
	2	12.89	14.52	13.27	13.02	13.87
	3		13.93	11.75	12.79	12.16
	Delta	0.45	3.61	2.59	0.75	1.71
	Rank	5	1	2	4	3
Toughness modulus (MPa)	1	12.319	11.089	11.071	10.815	10.938
	2	8.066	9.399	9.576	9.849	9.941
	3	–	10.090	9.931	9.914	9.699
	Delta	4.253	1.690	1.495	0.966	1.239
	Rank	1	2	3	5	4
Failure strain	1	0.05834	0.05570	0.05077	0.05298	0.05299
	2	0.04523	0.04874	0.05036	0.05194	0.05066
	3	–	0.05091	0.05422	0.05042	0.05170
	Delta	0.01311	0.00696	0.00386	0.00256	0.00233
	Rank	1	2	3	4	5

#### S/N ratio

**Fig. 15:**
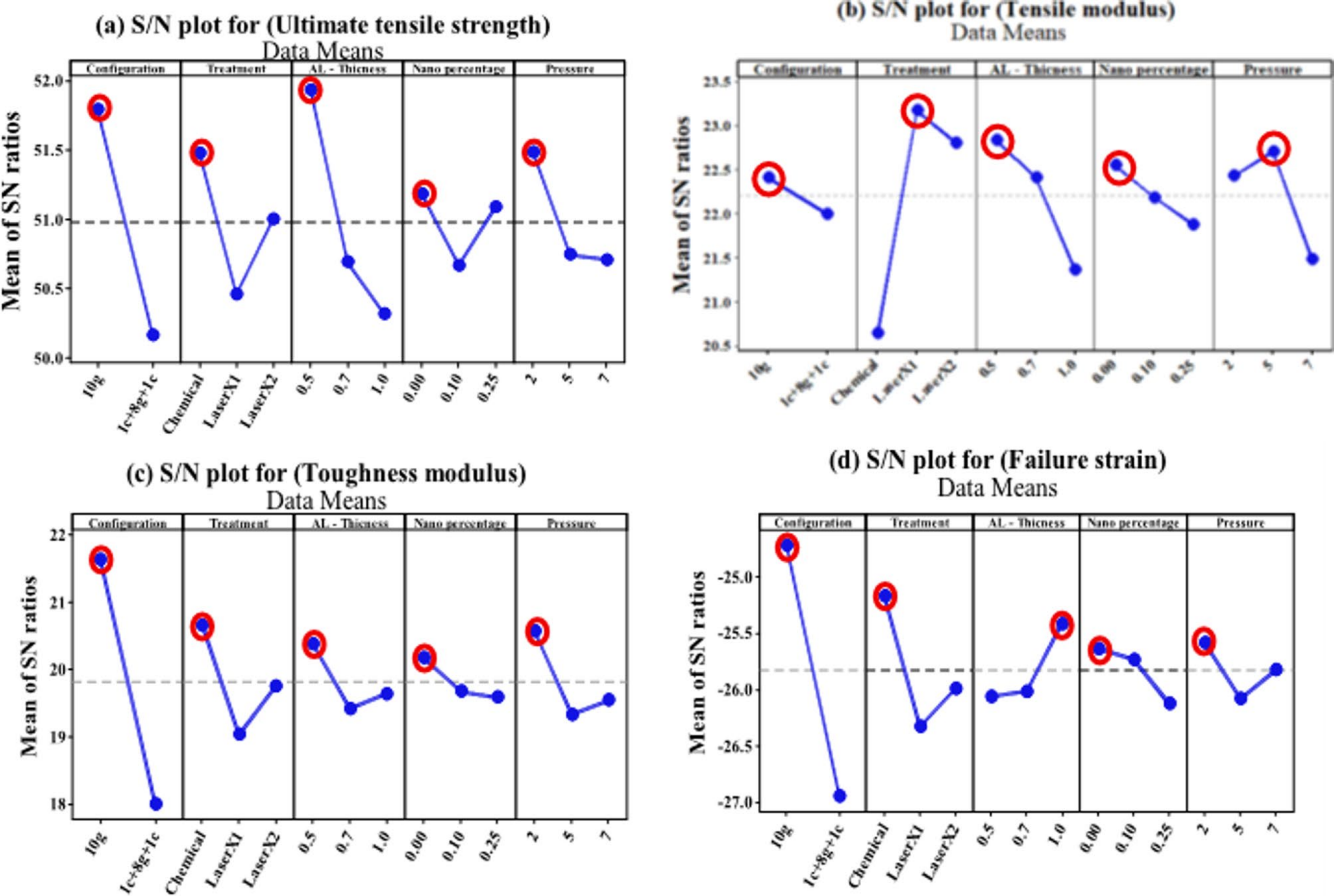
Signal/noise ratio (S/N) graph for (**a**), Ultimate tensile strength (**b**), Tensile modulus, (**c**) Toughness modulus, and (**d**) Failure strain.

Figure 15 shows the main effect plots of the signal-to-noise ratio (S/N) for four measured mechanical properties: for ultimate tensile strength, tensile modulus, toughness modulus and failure strain. In the Taguchi method, the signal-to-noise ratio (S/N) is considered a critical tool used to determine the robustness of the manufacturing process. It combines the average performance and the variation (or “noise”) on a single scale. For this investigation, where the goal is to achieve maximum performance of mechanical properties, the S/N property “the larger the better” was used. Therefore, the factor levels that achieve the highest signal-to-noise ratio are considered ideal, as they not only produce the best performance but also the most consistent and reliable results with the least variation.

When analyzing Fig. 15a, the signal-to-noise ratio (S/N) for tensile strength shows a significant dependence on the configuration factor. The significantly higher signal-to-noise ratio (S/N) for level 1 (full glass composition ‘10 g’) confirms the conclusion from the mean response analysis and indicates that this composition produces tensile strength values that are not only higher on average but also more reliable and repeatable. The slopes for aluminum thickness and treatment also indicate that these factors significantly affect the durability of tensile strength, as Level 1 for aluminum thickness (0.5 mm) and chemical treatment (level 1) produce more stable results. The relatively flat line for nano percentage indicates that, despite its impact on average strength, its contribution to result consistency is minimal.

The S/N diagram for the tensile modulus shown in Fig. 15b demonstrates a clear relationship. The factor levels with maximum signal-to-noise ratio are level 2 for surface treatments (a laser 1 mm scanning texture) and level 1 for the aluminum thickness of 0.5 mm; this is consistent with the average response analysis but differs by the important factor that the levels of those factors provide the most stable and reliable coefficient values. The fact that the lines for a few factors are not flat (e.g., processing, nano ratio) reveals that the variation of the modulus is dependent on the level chosen, if less so in terms of average effect. Therefore, to maximize high modulus and reliability, laser 1 mm scanning texture and thinner aluminum of 0.5 mm layers are ideal.

Figure 15c for toughness modulus shows the most dramatic contrast, reflecting the average response data. The signal-to-noise ratio for Level 1 of the configuration factor far exceeds Level 2. This evidence strongly confirms that the full fiberglass laminate (‘10 g’) is the uncontested most robust choice for maximizing energy absorption, resulting in outcomes with the highest average and lowest variance. Other factors have a relatively minor impact on the consistency of durability.

Lastly, Fig. 15d for failure strain has a similar trend to toughness, which would be expected since strain is an important component of toughness. Once again, the all-glass configuration (Level 1) gave the best S/N ratio, indicating its contribution to superior and consistent ductility. The graph also suggests that there are differences between aluminum surface treatments in affecting the consistency of the failure strain.

#### Analysis of variance (ANOVA)

The results obtained by the Taguchi method were verified with a statistical analysis of variance (ANOVA) on the tensile properties studied: ultimate tensile strength, elastic modulus, toughness modulus, and failure strain. Results of analysis of variance were used to evaluate relative contribution and significance of considered manufacturing parameters. The effect of the laminate configuration on the tensile performance of the fabricated graphene-enhanced fiber metal laminates is shown in Table 6, which was the most important factor. For the ultimate tensile strength, the laminate configuration contributed 32.17% to the total variance and was statistically significant (*P* = 0.020). Likewise, surface treatments significantly contributed to the observed variance in the elastic modulus (18.77%) (*P* = 0.020). As Table 6 also indicates, the effect of laminate configuration on the modulus of toughness and strain to failure was more pronounced, explaining 56.99% and 64.28% of the variance, respectively. Furthermore, significant effects of laminate configuration on the modulus of toughness (*P* = 0.003) and strain to failure (*P* < 0.001) were found, supporting the fact that laminate configuration is the most critical parameter for determining the energy absorption and deformation mode of the studied laminates. The ANOVA results are in strong agreement with the Taguchi analysis and consistently demonstrate that laminate configuration is the primary factor affecting the tensile performance of the fabricated laminates. This statistical assessment further confirms the reliability of the Taguchi-based optimization approach adopted in this study.

**Table 6:** Summary of the ANOVA results for the investigated tensile properties.

Tensile property	Dominant factor	Contribution (%)	*P*-value
Ultimate tensile strength	Configuration	32.17	0.020
Tensile modulus	Surface treatment	45.61	0.011
Toughness modulus	Configuration	56.99	0.003
Failure strain	Configuration	64.28	< 0.001

## Validation of experiments

After determining the optimal values for the design parameter, a validation experiment is performed at the optimal values as the next step in the Taguchi method. This step is performed as a confirmation and verification tool for the accuracy of the optimization results. By performing the validation experiment, the improvements that can be made in the tensile properties can be evaluated against the corresponding experiment results. This process ensures that the parameters selected improve the tensile properties, thereby verifying the optimization process. Additionally, any differences between the predicted values and the experiment values can be analyzed to improve the model and make the optimization methodology more reliable. A confirmation experiment was required for this study, as the optimal values for the parameter combinations were glass fiber configuration, chemical surface treatment, aluminum thickness of 0.5 mm, 0% graphene nanoparticles, and curing pressure of 2 bar, accomplishing minimum variance around the target value for ultimate tensile strength and toughness modulus. And this goal is achieved in Experiment 1, as shown in Table 2. Optimal values for the design parameter for tensile modulus are a laser 1 mm scanning texture, glass fiber laminate configuration, aluminum thickness of 0.5 mm, 0% graphene nanoparticles, and a curing pressure of 5 bar this goal is achieved in Experiment 4. Also, Optimal values for the design parameter for failure strain are glass fiber configuration, chemical surface treatment, aluminum thickness of 1 mm, 0% graphene nanoparticles, and curing pressure of 2 bar. The orthogonal array design did not result in optimal combinations for failure strain, as shown in Table 2. Therefore, the experimental values were compared with the expected values of ultimate tensile strength, tensile modulus and toughness to evaluate the precision of the optimization process and the reliability of the predictive model in predicting the tensile response for the specimens fabricated with the selected process parameters. Table 7 shows the differences between experimental and predicted values for ultimate tensile strength, tensile modules and toughness modules. The differences between the predicted and experimentally obtained values for ultimate tensile strength, tensile modulus and toughness modules are 2.7%,1.75% and 1.2%, respectively. The predicted responses closely matched the measured responses, thus confirming that the models were reliable, as the percentage error for each response was less than 5%, which is considered an acceptable range of statistical reliability [54]. Therefore, the results obtained from the confirmation tests reflect successful optimization. There are various reasons for the difference between the experimental values and the predicted values, which are called “random errors.” The random error could arise from various sources, such as variability inherent to the hand-layup process, which may have introduced inconsistencies in fiber alignment and resin distribution, while non-uniform graphene dispersion across the matrix remains a possibility despite the use of ultrasonication. Additionally, pressure distribution across the laminate during compression molding was not guaranteed to be perfectly uniform. Surface treatment quality likely varies between specimens, particularly with respect to etch depth uniformity and laser power consistency. Finally, environmental conditions during the 24-hour room temperature cure, including fluctuations in laboratory temperature and humidity, may have further contributed to specimen-to-specimen variability. On the other hand, theoretical calculations generally consider ideal conditions for all parameters and components used in the process, which makes the results entirely different from the actual values measured during experimentation. For example, theoretical calculations typically assume an ideal bonding condition between the materials’ components and the other structural components, as well as the absence of cavities in the specimen’s structure that may arise during the fabrication process. However, achieving an ideal bonding condition and a completely void-free structure is not always possible due to limitations associated with the process and the properties of the materials used.

**Table 7:** Experimental and predicated results for confirmation test specimens.

Tensile property	Experimental	Predicated	Error (%)
Ultimate tensile strength (MPa)	472.12	485.30	2.7
Tensile modules (GPa)	17.44	17.134	1.75
Toughness modulus (MPa)	15.27	15.46	1.2

## Comparative study of tensile properties for FMLs laminates

As shown in Table 8, results of the present study are broadly consistent with published data for glass fiber aluminum laminate types and hybrid FMLs. The ultimate tensile strength range is from 254 to 472 MPa and tensile modulus range from 7.7 to17.4 GPa compare favorably with comparable FMLs. The lower end of the ultimate tensile strength range reflects specimens with suboptimal parameter combinations as Experiments 5 and 1, while the upper end as Experiment 1 of 472 MPa that is competitive with the best reported values for glass fiber configuration with chemical surface treatment.

**Table 8:** Comparative study of tensile properties for FMLs laminates.

Reference	Material system	Surface Treatment	Ultimate tensile strength (MPa)	Tensile modulus (GPa)	Toughness (MPa)	Key variable
Megahed et al.^15^	Glass fiber aluminum laminate	Phosphoric acid anodizing	310–420	14–20	8–14	Nanofiller type
Belkondi et al.^9^	Carbon fiber aluminum laminate	Mechanical abrasion, nitric acid etching, P2 etching, sulfuric acid anodizing (SAA), and electric discharge machine	340–490	20–28	Not reported	Surface treatment
Hosseini Abbandanak et al.^17^	Carbon fiber aluminum laminate	Alkaline + acid etching	250–380	15–21	7–13	Graphene addition
Askin & Turen. ^18^	Carbon fiber Aluminum Laminate	Phosphoric acid anodizing	370–510	22–31	Not reported	Graphene addition
Zakaulla. ^16^	Glass fiber aluminum laminate	Mechanical abrasion	290–460	16–24	6–13	Graphene addition
Present Study	Al treatment/E-glass & C-glass/GNP (0–0.25 wt%)/pressure (2–7 bar)/Al thickness	HCl/NaOH & Laser (1 mm, 2 mm spacing)	254–472	7.7–17.4	5.6–15.3	5-param Taguchi optimization

The toughness modulus range is from 5.6 to 15.3 MPa that is consistent with values reported by Megahed et al.[55] and Hosseini Abbandanak et al. [56]. Unlike previous investigations that mainly concentrated on a single parameter, the current work simultaneously optimized five processing parameters using the Taguchi approach. The suggested hybrid FMLs configuration’s potential for advanced lightweight structural applications was demonstrated by this thorough optimization technique achieving a balanced combination of strength, stiffness, strain and toughness.

## Conclusion

The current study evaluated the impact of five critical processing variables on the tensile properties of fiber metal laminates produced through hand layup and compression. The effects of laminate configuration, aluminum surface treatment, aluminum sheet thickness, graphene nanoplatelet content, and curing pressure on four important mechanical responses, ultimate tensile strength, tensile modulus, toughness modulus, and failure strain, were systematically studied using a Taguchi L18 orthogonal array. Analysis of variance (ANOVA) was used to evaluate the relative contribution of each manufacturing parameter to the overall observed variation in mechanical performance. Signal-to-noise (S/N) ratio analysis was used to evaluate the robustness of the process to variability in the experiments. Based on the comprehensive results of the experiments, the following main conclusions were drawn:

1. The results indicated that all five manufacturing parameters studied, which are laminate configuration, aluminum surface treatment, aluminum sheet thickness, graphene nanoplatelet (GNP) content, and curing pressure, are the controlling parameters for the tensile properties of the produced FMLs specimens. Nevertheless, their relative contributions changed significantly across the four mechanical responses ($\:{\upsigma\:}$_ult_, *E*, U_T_, and $\:\epsilon\:$_f_), confirming that accurate and property-specific control of process variables is needed to obtain the desired mechanical behavior in FMLs systems.
2. Laminate configuration was the most significant factor controlling $\:{\upsigma\:}$_ult_, accounting for 32.17% of the total variance (*P* = 0.020). The tensile strength of the all-glass fiber configuration (10 g) was consistently higher than that of the hybrid glass–carbon counterpart (1c + 8 g + 1c) with a maximum mean $\:{\upsigma\:}$_ult_ of 472.12 MPa.
3. The most important contributor to the variance in the tensile modulus E was the aluminum surface treatment, which accounted for 45.61% of the total variance (*P* = 0.011). This observation shows the crucial role of the quality of interfacial adhesion at the metal composite interface to control the stiffness of laminates.
4. In general, the toughness modulus U_T_ was mainly influenced by the laminate configuration, accounting for 56.99% of the total variance (*P* = 0.003). The only glass configuration gave a maximum mean U_T_ of 15.27 MPa.
5. Failure strain $\:\epsilon\:$_f_ was most sensitive to the laminate configuration, contributing to 64.28% of the total variance (*P* < 0.001). The second most influential factor was surface treatment (Δ = 0.00696) and then aluminum thickness (Δ = 0.00386). The effects of GNP content and curing pressure on ductility were relatively small.
6. The optimal parameter combination for maximizing $\:{\upsigma\:}$_ult_ and U_T_ was identified as the following: all-glass fiber configuration (10 g), chemical surface treatment, aluminum thickness of 0.5 mm, 0 wt% graphene nanoparticles, and curing pressure of 2 bar. The best optimal parameters for maximizing E were a laser-textured surface with a scanning spacing of 1 mm, the glass fiber configuration, 0.5 mm aluminum thickness, 0% GNP content, and a curing pressure of 5 bar. The optimal parameters for maximizing $\:\epsilon\:$_f_ were glass fiber configuration, chemical surface treatment, 1.0 mm aluminum thickness, 0% GNP content, and a curing pressure of 2 bar.
7. The prediction errors for $\:{\upsigma\:}$_ult_, E, and U_T_ in the confirmation experiments at the optimal parameter settings were 2.7%, 1.75%, and 1.2%, respectively. All errors fell within the acceptable statistical reliability limit of 10%. This confirms the predictive capability and robustness of the Taguchi-based optimization model that was developed.

The results illustrated that the optimization of the parameters significantly improved the mechanical performance of FMLs, offering excellent guidance in producing high-performance FMLs as lightweight, damage-tolerant materials for aerospace and automotive applications where structural integrity is necessary in a lightweight structure.

## Data Availability

The datasets used during the current study are available from the corresponding author on reasonable request.
